# *In Vivo* Genome-Wide Gene Expression Profiling Reveals That *Haemophilus influenzae* Purine Synthesis Pathway Benefits Its Infectivity within the Airways

**DOI:** 10.1128/spectrum.00823-23

**Published:** 2023-05-17

**Authors:** Begoña Euba, Celia Gil-Campillo, Javier Asensio-López, Nahikari López-López, Emel Sen-Kilic, Roberto Díez-Martínez, Saioa Burgui, Mariette Barbier, Junkal Garmendia

**Affiliations:** a Instituto de Agrobiotecnología (IDAB), Consejo Superior de Investigaciones Científicas (CSIC)-Gobierno de Navarra, Mutilva, Spain; b Centro de Investigación Biomédica en Red de Enfermedades Respiratorias (CIBERES), Madrid, Spain; c Asociación de la Industria Navarra (AIN)-Gobierno de Navarra, Cordovilla, Spain; d Vaccine Development Center, West Virginia University Health Sciences Center, Morgantown, West Virginia, USA; e Department of Microbiology, Immunology and Cell Biology, West Virginia University School of Medicine, Morgantown, West Virginia, USA; f Telum Therapeutics, Noain, Spain; g Conexión Nanomedicina-CSIC, Madrid, Spain; Forschungszentrum Jülich GmbH

**Keywords:** *Haemophilus influenzae*, airway infection, *in vivo* RNA-seq, metabolic requirements, purine synthesis, purine analogs, lipooligosaccharide decoration, natural competence

## Abstract

Haemophilus influenzae is a human-adapted bacterial pathogen that causes airway infections. Bacterial and host elements associated with the fitness of H. influenzae within the host lung are not well understood. Here, we exploited the strength of *in vivo*-omic analyses to study host-microbe interactions during infection. We used *in vivo* transcriptome sequencing (RNA-seq) for genome-wide profiling of both host and bacterial gene expression during mouse lung infection. Profiling of murine lung gene expression upon infection showed upregulation of lung inflammatory response and ribosomal organization genes, and downregulation of cell adhesion and cytoskeleton genes. Transcriptomic analysis of bacteria recovered from bronchoalveolar lavage fluid samples from infected mice showed a significant metabolic rewiring during infection, which was highly different from that obtained upon bacterial *in vitro* growth in an artificial sputum medium suitable for H. influenzae. *In vivo* RNA-seq revealed upregulation of bacterial *de novo* purine biosynthesis, genes involved in non-aromatic amino acid biosynthesis, and part of the natural competence machinery. In contrast, the expression of genes involved in fatty acid and cell wall synthesis and lipooligosaccharide decoration was downregulated. Correlations between upregulated gene expression and mutant attenuation *in vivo* were established, as observed upon *purH* gene inactivation leading to purine auxotrophy. Likewise, the purine analogs 6-thioguanine and 6-mercaptopurine reduced H. influenzae viability in a dose-dependent manner. These data expand our understanding of H. influenzae requirements during infection. In particular, H. influenzae exploits purine nucleotide synthesis as a fitness determinant, raising the possibility of purine synthesis as an anti-H. influenzae target.

**IMPORTANCE** *In vivo*-omic strategies offer great opportunities for increased understanding of host-pathogen interplay and for identification of therapeutic targets. Here, using transcriptome sequencing, we profiled host and pathogen gene expression during H. influenzae infection within the murine airways. Lung pro-inflammatory gene expression reprogramming was observed. Moreover, we uncovered bacterial metabolic requirements during infection. In particular, we identified purine synthesis as a key player, highlighting that H. influenzae may face restrictions in purine nucleotide availability within the host airways. Therefore, blocking this biosynthetic process may have therapeutic potential, as supported by the observed inhibitory effect of 6-thioguanine and 6-mercaptopurine on H. influenzae growth. Together, we present key outcomes and challenges for implementing *in vivo*-omics in bacterial airway pathogenesis. Our findings provide metabolic insights into H. influenzae infection biology, raising the possibility of purine synthesis as an anti-H. influenzae target and of purine analog repurposing as an antimicrobial strategy against this pathogen.

## INTRODUCTION

Haemophilus influenzae is a human-adapted Gram-negative bacterial pathogen. Successful introduction of the H. influenzae type B (Hib) vaccine has driven its almost complete disappearance in countries with established child immunization programs, while nontypeable H. influenzae (NTHi) strains cause otitis media, conjunctivitis, sinusitis, and lower respiratory infections in children; exacerbations of chronic obstructive pulmonary disease (COPD) and cystic fibrosis in adults; and invasive disease in neonates, immunocompromised adults, and the elderly (1–4). One major gap in understanding NTHi infection is the lack of detailed information about molecular interaction networks between pathogen and host at the infection site. Moreover, H. influenzae antimicrobial resistance shows an overall increasing trend (5), and target identification for drug discovery should contribute to expand the global antibacterial pipeline. In this context, delineation of this host-pathogen interplay by applying -omic approaches on suitable *in vivo* model systems of infection paves the way to informing the development of new therapeutics.

Pioneer methodologies have been developed for genome-wide screening of H. influenzae genes required in the lower airway using a murine model of lung infection (6, 7). Later, a chinchilla model of otitis media contributed to identifying global changes in gene, protein, and metabolite profiles during disease (8, 9). Also, cell-to-cell leukotriene B4 signaling between cells was reported by single-cell RNA sequencing in a murine middle ear model infected with NTHi (10). However, -omic studies remain mostly focused on *in vitro* H. influenzae biofilm communities, where the combination of proteome, transcriptome, and metabolome analyses revealed key bacterial metabolic traits (11, 12). The relevance of bacterial metabolism was also observed by dual transcriptome sequencing (RNA-seq) gene expression profiling during NTHi infection of ciliated human bronchial epithelial cells or monocyte-derived macrophages (13, 14). In parallel, profiling of host mRNA signatures upon H. influenzae infection revealed dysregulation of the target cell cytoskeleton, particularly the intermediate filament network and junctional complexes in bronchial epithelia (14), and specific enrichment of intracellular immune response pathways in macrophages (13).

Overall, the current lack of *in vivo*-omic studies allowing for broad multifactorial analyses limits our understanding of the H. influenzae host-pathogen interplay within the host airways. Here, we used RNA-seq on a previously used murine model system of H. influenzae airway infection (15–18). Our data shed light on the murine lung global responses to H. influenzae infection, with upregulation of inflammatory response and ribosomal organization gene expression and downregulation of cell adhesion and cytoskeleton gene expression. We also disclose bacterial metabolic requirements during infection, where a range of transport systems, together with purine nucleotide and non-aromatic amino acid biosynthesis pathways, seem to contribute to overcoming nutritional limitations within the lung environment. *In vivo* bacterial gene expression profiling was not comparable to that obtained *in vitro* using a synthetic medium designed to model host sputum conditions (19). These discrepancies limited its suitability to model the *in vivo* conditions analyzed in this study. Importantly, the association between *in vivo* upregulation of bacterial gene expression and mutant attenuation led us to assess the therapeutic potential of targeting the H. influenzae genes/pathways that are upregulated upon infection. This notion was supported by the bacterial growth-inhibitory effect of purine analogs, further promoting purine analog repurposing for use as an antibacterial against this pathogen.

## RESULTS

### Airway infection triggers host transcriptional reprogramming: global gene expression analyses of the murine lung.

**FIG 1:**
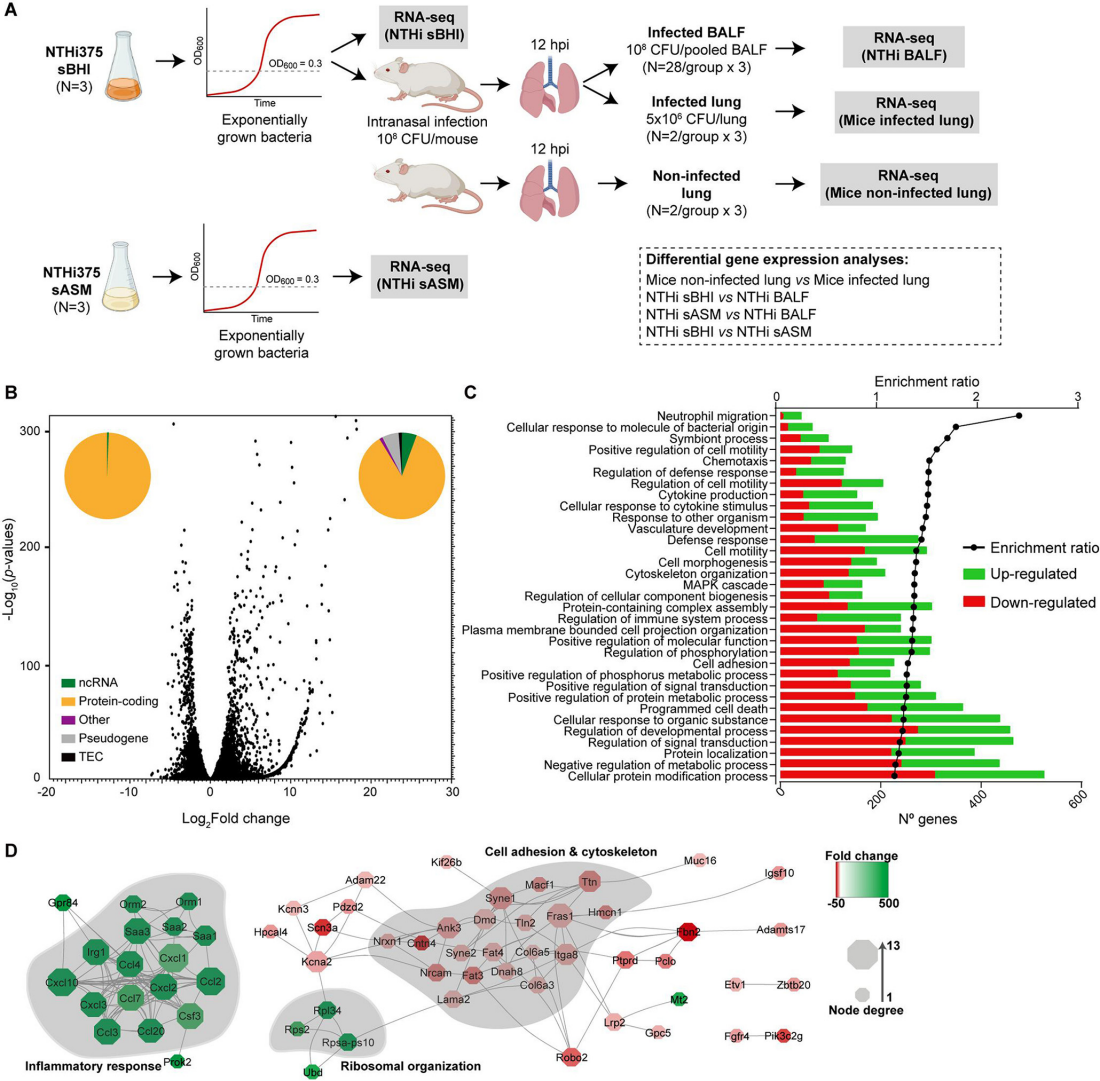
Study design and Mus musculus global lung expression profile upon infection by Haemophilus influenzae. (A) Schematic representation of sample generation for RNA-seq. NTHi375 was exponentially grown in sBHI and used (i) to generate NTHi sBHI samples and (ii) to infect mice and generate NTHi BALF samples. In addition, lung homogenates from infected and uninfected mice were processed to generate mouse infected and non-infected lung samples, respectively. NTHi375 was also exponentially grown in sASM to generate NTHi sASM samples. All samples were processed for RNA-seq. Analyses of differential gene expression were carried out by the following comparisons: mouse non-infected versus infected lung samples; NTHi sBHI versus NTHi BALF samples; NTHi sASM versus NTHi BALF samples; and NTHi sBHI versus NTHi sASM samples. (B) Characterization of *M. musculus* global expression profile upon airway infection. Volcano plot represents the fold changes of murine differentially expressed (DE) genes in mouse lungs upon H. influenzae infection. Pie chart shows the percentage of protein-coding, noncoding RNA, pseudogenes, TEC [to be experimentally confirmed; refers to areas of the genome that are described by Ensembl as clusters which have poly(A) features that could indicate the presence of protein-coding genes], and other DE genes upon H. influenzae infection. (C) Ingenuity Pathway Analysis (IPA) of canonical pathways that are significantly up- (green) or downregulated (red) in mice during H. influenzae infection. (D) STRING analyses of DE mouse genes upon H. influenzae infection representing inflammatory response, ribosomal organization, and cell adhesion and cytoskeleton pathways. Node size represents the degree of connectivity of each gene in the network. Node colors are based on the data set’s differential regulation of each gene (upregulated, green; downregulated, red).

Transition from the environment to a host triggers vast changes in gene expression in bacterial pathogens (20–24). To gain insights into how H. influenzae adapts to the host environment during this transition, we performed RNA-seq of the pathogen and host during acute lung infection in mice. A schematic representation of the experimental design is shown in Fig. 1A. NTHi375 bacterial strain was grown in brain heart infusion supplemented with hemin and NAD (sBHI) at 37°C and 10^8^ CFU were used to infect CD1 mice by intranasal administration. Lungs from infected animals were collected at 12 h postinfection (hpi) for RNA purification. As controls, RNA was purified from non-infected animals and from NTHi375 grown in sBHI. For all samples, rRNA was depleted, and three libraries were prepared and sequenced. Read data were mapped to H. influenzae and Mus musculus genomes independently using CLC Genomics. A total of 70 to 75 million 2 × 150-bp reads were obtained for each sample. Differential gene expression analysis highlighted 2,078 upregulated versus 2,229 downregulated genes in infected compared to non-infected lungs (Data Set S1 in the supplemental material). The vast majority of the genes with statistical changes in expression were protein-coding genes (Fig. 1B). Strikingly, 5.6% of the upregulated genes in the lung in response to infection were predicted to encode noncoding RNAs. The STRING and GO Term analysis showed that genes whose products are involved in inflammatory responses were upregulated during infection. These genes are associated with biological Gene Ontology (GO) terms such as neutrophil chemotaxis, regulation of defense response, and cytokine production. Among these, chemokines and genes encoding serum amyloid A were the most differentially upregulated protein-encoding genes. Differential gene expression compatible with the remodeling of extracellular matrix proteins (ECM) following NTHi infection was also observed (Data Set S1). On the other hand, genes associated with cell adhesion and cytoskeleton were downregulated (Fig. 1C and D). In summary, dysregulation of the murine lung cytoskeleton, ECM remodeling, and pro-inflammatory responses were observed upon H. influenzae infection.

### The *H. influenzae* murine airway infection transcriptome is distinct from *in vitro* models.

To gain insights into the bacterial transcriptome during infection, the reads obtained from the infected lung were mapped to the pathogen using CLC Genomics. Unfortunately, only approximately 0.25% of the reads in each sample mapped to the pathogen, indicating that the amount of bacterial RNA in the infected tissue was too low for analysis. These data indicate that the methodology described above provides RNA in sufficient quantities to study the transcriptome of the host, but not that of the pathogen in the same sample. Under the conditions tested, approximately 3 × 10^6^ CFU/lung were quantified. To increase bacterial RNA yields from *in vivo* samples, mice were infected (see above), bacteria recovered from bronchoalveolar lavage fluid (BALF) samples were pooled, reaching up to ~1 × 10^8^ CFU/pooled BALF sample, and total RNA was purified (see Materials and Methods). After rRNA depletion, library preparation, and Hi-Seq sequencing, 150 to 179 million 2 × 150-bp reads were obtained for each BALF sample. Read mapping to the pathogen was greater than in lung tissue, with 13.4% to 15.2% of the reads mapped in pairs to the pathogen. These data indicate that this methodology provides quality RNA in sufficient quantities for *in vivo* profiling of the H. influenzae transcriptome.

**FIG 2:**
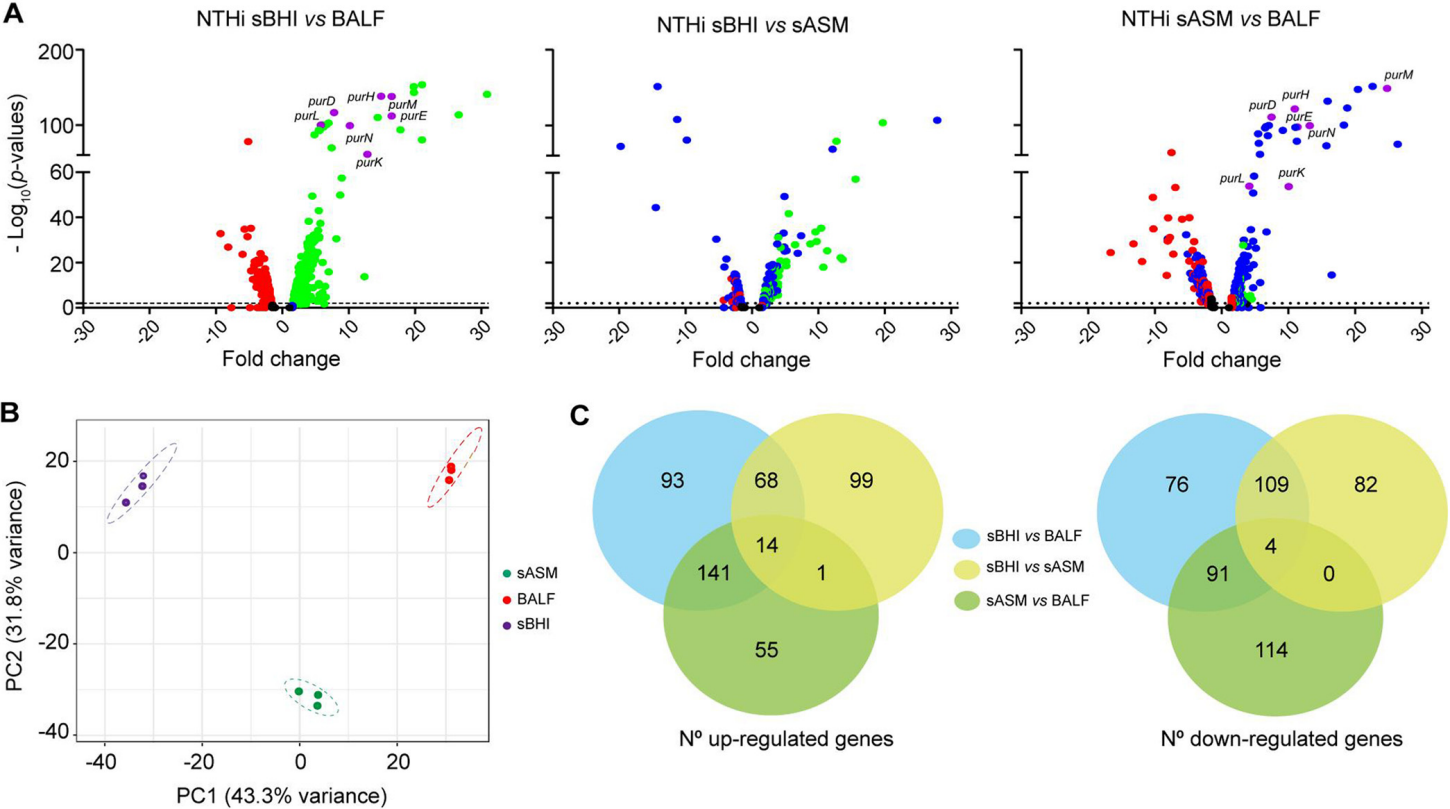
Characterization of H. influenzae global expression profile *in vitro* and upon airway infection. (A) Volcano plots represent the fold change of bacterial DE genes upon murine airway infection (NTHi BALF) compared to bacterial growth *in vitro*, in sBHI (left panel), or in sASM (right panel); volcano plot represents the fold change of bacterial DE genes upon growth in sASM compared to growth in sBHI (middle panel). Dot colors indicate differential expression for each bacterial gene in each comparison: upregulated, green; downregulated, red (i.e., in the left panel, green dots indicate NTHI375 genes upregulated in BALF versus sBHI samples). Blue dotted genes in middle and right panels indicate those that were also found when comparing NTHi sBHI versus BALF samples. Purple dotted genes indicate those involved in purine synthesis (*pur* genes), commonly upregulated in the right (NTHi sASM versus BALF) and left (NTHi sBHI versus BALF) panel comparisons. Not-shown outlier in middle panel, fold change value of −501.7 (merP_1, heavy-metal-associated domain-containing protein); not-shown outlier in right panel, fold-change value of +67,4 (*ftsK*, DNA translocase). (B) Principal-component analysis plot of pathogen transcriptional response upon *in vitro* (sBHI or sASM) growth or *in vivo* airway infection (BALF). Replicates cluster closely together, and bacterial transcriptional responses are different under the three tested conditions. (C) Venn diagrams show the number of bacterial DE genes in the three analyses shown in panel A. Gene identities are shown in Data Set S2. Left panel, upregulated genes; right panel, downregulated genes. Analysis-specific and commonly found numbers of genes are indicated in black. Upregulated genes: 14 genes were commonly upregulated in all three analyses; 93, 99, and 55 genes were specific to the sBHI versus BALF, sBHI versus sASM, and sASM versus BALF comparisons, respectively; 82 (68 + 14) were commonly upregulated in the sBHI versus BALF and sBHI versus sASM analyses; 15 (14 + 1) commonly upregulated in the sASM versus BALF and sBHI versus sASM analyses; and 155 (141 + 14) genes commonly upregulated in the sBHI versus BALF and sASM versus BALF analyses. Downregulated genes: 4 genes were commonly downregulated in all three analyses; 76, 82, and 114 genes were specific to the sBHI versus BALF, sBHI versus sASM, and sASM versus BALF comparisons, respectively; 113 (109 + 4) were commonly downregulated in the sBHI versus BALF and sBHI versus sASM analyses; 4 (4 + 0) were commonly downregulated in the sASM versus BALF and sBHI versus sASM analyses; and 95 (91 + 4) were commonly downregulated in the sBHI versus BALF and sASM versus BALF analyses.

**FIG 3:**
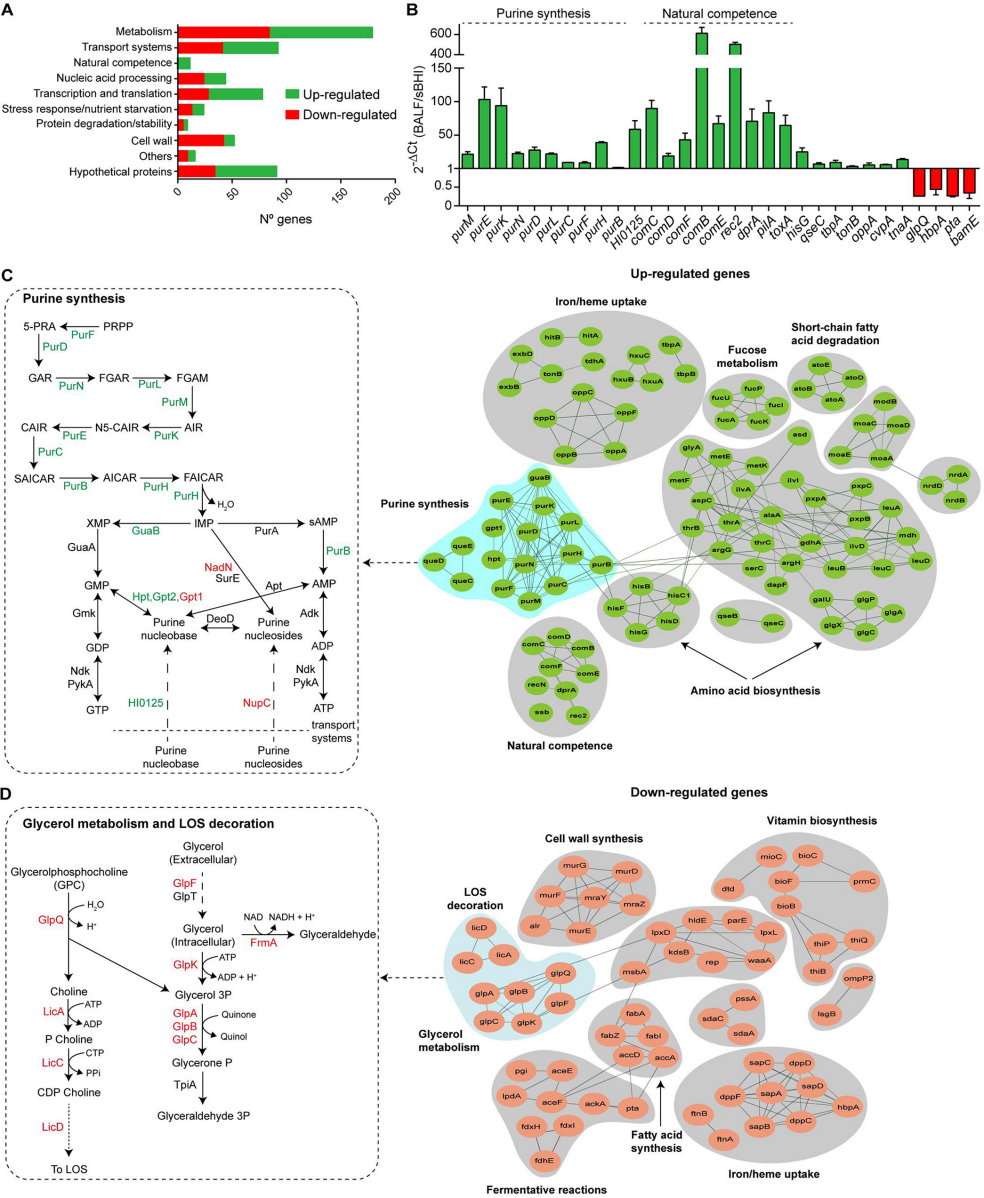
H. influenzae global gene expression profiling upon airway infection. (A) Number of H. influenzae genes that were significantly up- (green) or downregulated (red) in murine BALF samples compared to sBHI growth, organized into functional categories. (B) Validation of selected up- (green) and downregulated (red) H. influenzae genes *in vivo* versus sBHI growth. NTHi375 was grown in sBHI and collected during the exponential phase; BALF samples were recovered from NTHi375-infected mice at 12 hpi. Purified RNA was used to determine the ratio of bacterial gene expression by RT-qPCR. Data are shown as mean ± standard deviation (SD). (C) STRING analysis of H. influenzae genes upregulated upon infection, showing purine synthesis, amino acid synthesis, iron/heme uptake-sensing, l-fucose metabolism, short-chain fatty acid degradation, natural competence, ribonucleoside-triphosphate reductases, and molybdopterin biosynthesis. Left panel: H. influenzae differentially expressed genes involved in purine synthesis. Green, reactions catalyzed by genes upregulated *in vivo*; red, reactions catalyzed by genes downregulated *in vivo*. PRPP, phosphoribosyl pyrophosphate; PRA, phosphoribosylamine; GAR, glycinamide ribonucleotide; FGAR, formylglycinamide ribonucleotide; FGAM, formylglycinamidine ribonucleotide; AIR, aminoimidazole ribonucleotide; CAIR, phosphoribosyl carboxyaminoimidazole; SAICAR, succinocarboxyamide carboxyaminoimidazole ribonucleotide; AICAR, aminoimidazole carboxamide ribonucleotide; FAICAR, formaminoimidazole carboxamide ribonucleotide; sAMP, adenylsuccinate; purine nucleobases: guanine, xanthine, hypoxanthine, and adenine; purine nucleosides: guanosine, xanthosine, inosine, and adenosine. (D) STRING analysis of H. influenzae genes downregulated upon infection showing LOS decoration and cell wall biosynthesis, glycerol metabolism, fatty acid synthesis, vitamin biosynthesis and transport, iron/heme uptake, and sugar fermentative reactions. Left panel: H. influenzae genes downregulated upon infection, involved in glycerol metabolism and LOS decoration with PCho. Red, reactions catalyzed by genes downregulated *in vivo*. Node colors in panels C and D are based on the data set’s differential regulation of each gene (upregulated, green; downregulated, red).

EDGE (Extraction of Differential Gene Expression) statistical analysis was used to measure differences in gene expression. A total of 316 H. influenzae genes were found to be upregulated and 280 were downregulated *in vivo* compared to sBHI *in vitro* growth with *P* < 0.05 (Data Set S2; Fig. 2A, left panel). This differential expression was validated by reverse transcription-quantitative PCR (RT-qPCR) for a subset of genes (Fig. 3B, Fig. S1).

Using BALF samples for *in vivo* bacterial transcriptomics may mostly profile transcriptomes from extracellular bacteria recovered upon sampling. Over the past decade, artificial sputum medium (ASM) models meant to mimic the environmental conditions experienced in the mucus have been valuable for studying Pseudomonas aeruginosa physiology (19). However, this type of synthetic medium failed to computationally classify Staphylococcus aureus transcriptomes as *in vivo* samples (25). ASM suitability for studying H. influenzae airway infection has not been previously tested. Here, the H. influenzae transcriptome was profiled upon growth in ASM which was adequately supplemented to allow bacterial growth (see Materials and Methods, Fig. S2). Exploratory analysis of our RNA-seq data sets for the H. influenzae transcriptome revealed transcriptional signatures unique to each of the three different environmental conditions (sBHI, BALF, sASM) as samples clustered independently (Fig. 2B).

Next, we used EDGE statistical analysis to measure differences in gene expression. We hypothesized that, if ASM supplemented with hemin and NAD (sASM) mimics BALF, differential gene expression would be comparable when analyzing sBHI versus BALF and sBHI versus sASM sample pairs. A total of 182 (68 + 99 + 1 + 14) H. influenzae genes were up- and 195 (109 + 82 + 4) were downregulated upon *in vitro* growth in sASM compared to sBHI, with *P* < 0.05 (Data Set S2; Fig. 2A, middle panel; Fig. S3; see Fig. 2C for gene number calculations). However, although 82 (68 + 14) and 113 (109 + 4) genes were commonly up- or downregulated in BALF and sASM compared to sBHI samples, 234 (93 + 141) and 167 (76 + 91) genes were exclusively up- or downregulated in BALF (Fig. 2C). Bacterial genes/pathways exclusively upregulated in BALF included purine, l-fucose, short-chain fatty acid, and amino acid metabolism, transport systems, natural competence machinery, and genes involved in stress response. Likewise, bacterial genes/pathways exclusively downregulated in BALF included glycerol, sugar and fatty acid metabolism, and lipooligosaccharide (LOS) decoration (Data Set S2, Fig. 3). Furthermore, if sASM mimics BALF, differential gene expression would be barely observed when comparing sASM and BALF samples. However, this was not the case, as a total of 211 (141 + 14 + 1 + 55) H. influenzae genes were found to be upregulated and 209 (91 + 4 + 114) were downregulated *in vivo* compared to sASM *in vitro* growth with *P* < 0.05 (Fig. 2A, right panel; Fig. 2C). In fact, a volcano plot representation of this analysis shows significant overlapping with that obtained when comparing sBHI versus BALF samples (Fig. 2A, right and left panels).

In summary, under the conditions tested, sASM did not mimic the murine airway environment used here for *in vivo* bacterial gene expression profiling, and was excluded from further analysis. We next focused on bacterial gene expression reprogramming upon infection by comparing NTHi sBHI (i.e., infecting inocula) and BALF samples.

### Expression of bacterial metabolic and surface-related genes differentiate *in vivo* and *in vitro* samples.

To define the systems which were differentially regulated under each experimental condition (sBHI and BALF), we analyzed data using GO term analysis of the biological processes, KEGG pathways, STRING analyses, and additional manual curation (see Materials and Methods; Fig. 3, Data Set S2). Our main findings are presented here.

**Changes in gene expression associated with bacterial metabolism.** A large proportion of genes differentially expressed during infection compared to sBHI growth encode products involved in bacterial metabolism (Fig. 3 and Fig. S1). Genes involved in amino acid biosynthesis (alanine, histidine, aspartate, threonine, leucine, isoleucine, valine, glycine, glutamate), fucose metabolism (*fucA*, *fucI*, *fucK*, *fucU*) and short-chain fatty acid degradation (*atoABDE*) pathways were upregulated. Notably, genes involved in purine—mostly *de novo* (*purF*, *purD*, *purN*, *purL*, *purM*, *purK*, *purE*, *purC*, *purB*, *purH*, *guaB*, *purB*), to a lower extent salvage (*hpt*, *gpt2*)—biosynthesis were also upregulated (Fig. 3C) (26). Conversely, genes involved in fatty acid biosynthesis (*accA*, *accD*, *fabA*, *fabI*, *fabZ*) were downregulated upon infection, next to genes involved in fermentative reactions leading to acetate production (*pta*, *ackA*, *fdnG*, *fdxH*, *fdxI*, *fdhE*); this may be unexpected given that *ackA* gene inactivation was previously shown to reduce fitness *in vivo* (18). Also, genes involved in glycerol metabolism (*glpK*, *glpA*, *glpB*, *glpC*), and glycerophosphorylcholine (GPC) transport and/or metabolism, including the GPC link with phosphorylcholine (PCho) decoration of the LOS molecule, were expressed at lower levels *in vivo* compared to sBHI tested conditions (*glpQ*, *lic*). This may also be the case for LOS sialylation, as expression of the *lsgB* and *lic3* genes was lower in BALF than in sBHI samples, and for LOS decoration with di-galactose residues (*lex1*/*lic2*). Downregulation of genes involved in bacterial cell wall synthesis was also observed for lipid A (*lpxD*, *msbA*, *lpxL*, *waaA*, *kdsB*) and peptidoglycan (*murDEFG*, *mraY*, *mraZ*) biosynthesis pathways (Data Set S2, Fig. 3D).

**Changes in bacterial gene expression associated with transport.** We also observed differential expression of a wide range of transport systems (Data Set S2, Fig. 3). As reported for other bacterial pathogens such as P. aeruginosa (23), the range of mechanisms used for iron and heme uptake were differentially regulated under each condition. The HbpA-Dpp and Sap heme uptake systems were upregulated *in vitro*; however, the OppABCDF heme, HitABC iron, TbpAB transferrin, and HxuCBA hemopexin uptake systems, together with TonB-ExbBD itself, were upregulated *in vivo*. Although complex due to redundancy of some of these systems (27, 28), these expression differences may relate to the sources of available iron encountered by NTHi in the host lung. Also related to iron sensing (29), the QseBC/FirRS system was upregulated *in vivo*. Regarding nucleobase transport, genes encoding purine (HI0125) and queuine (*yhhQ*) transporters were upregulated, while the nucleoside transporter encoding gene *nupC* was downregulated *in vivo*. Downregulation of glycerol transport *in vivo* may also align with glycerol-GPC metabolic processes linked to PCho decoration. Lastly, upregulation of fucose (*fucP*), arginine (*artP*) and serine/threonine (*sstT*) transport- and downregulation of serine transport (*sdaC*)-encoding genes was observed.

In conclusion, under the conditions tested, the infectious process stimulates H. influenzae purine and non-aromatic amino acid biosynthesis, and a wide range of transporters adapting to the local nutrient availability; on the other hand, we observed downregulated expression of genes involved in fatty acid synthesis, sugar fermentation, and bacterial cell wall synthesis pathways.

**Changes in expression of H. influenzae genes associated with bacterial natural competence.** H. influenzae actively transports environmental DNA fragments across the cell envelope into the cytoplasm. Incoming DNA fragments provide access to the nutrients in extracellular DNA, or recombine and replace homologous segments of the bacterial chromosome (30). In a previous study, Redfield et al. characterized gene expression changes during competence development *in vitro* by sampling H. influenzae during exponential growth in sBHI and after transition to the starvation medium MIV, suitable for developing natural competence (31). This study defined the competent regulatory element (CRE), later renamed the Sxy-dependent cyclic AMP receptor protein (CRP-S) regulon, which consists of 26 genes organized in 13 operons and is strongly upregulated by MIV nutrient starvation. Seventeen of these genes are necessary for *in vitro* natural transformation in MIV (32).

**FIG 4:**
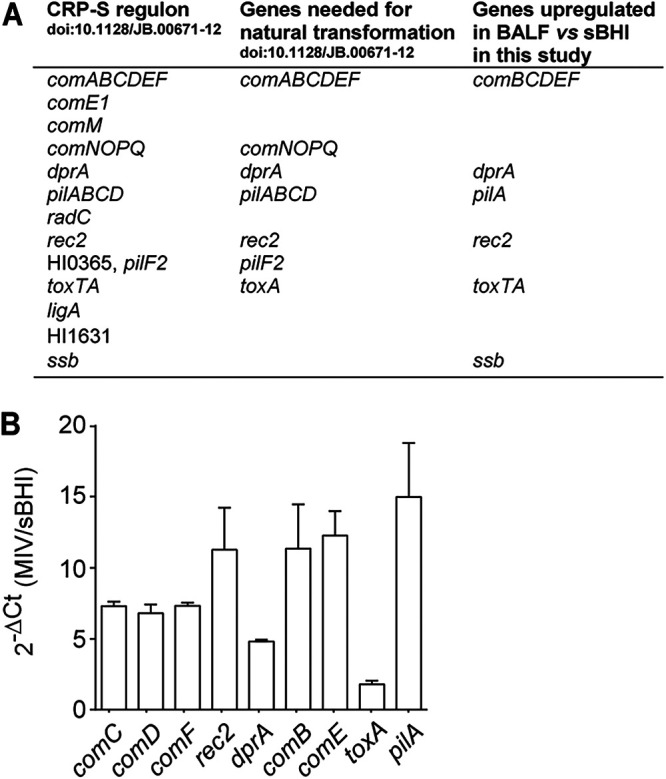
H. influenzae genes belonging to the natural competence machinery are upregulated *in vivo*. (A) A correlation between genes previously established to belong to the competence CRP-S regulon (31), those previously established to be absolutely required for natural transformation *in vitro* (32), and those shown to be upregulated *in vivo* in this study is detailed. (B) NTHi375 was grown in sBHI or MIV and collected during the exponential phase (see Supplementary Methods in Supplemental File 3). A fold change in gene expression was determined by RT-qPCR. Data are shown as mean ± standard error of the mean (SEM).

Our data showed *in vivo* upregulation of 11 genes belonging to the CRP-S regulon, *comBCDEF*, *dprA*, *pilA*, *rec2*, *toxTA*, and *ssb*; 9 of these are required for natural transformation *in vitro*, i.e., *comBCDEF*, *dprA*, *pilA rec2*, and *toxA*. In contrast, overexpression of the *comNOPQ* and *pilF2* genes, also needed for natural transformation in MIV medium, was not observed (Data Set S2, Fig. 3A to C, Fig. 4A). Also, the extent of NTHi375 bacterial differential gene expression between BALF and sBHI samples was higher than when comparing MIV- and sBHI-grown bacterial cultures (Fig. 3B and 4B). Based on these data, we hypothesized that the observed gene upregulation may not induce natural competence within the airways, as several key players are not overexpressed. Alternatively, natural competence requirements *in vivo* may differ from those *in vitro*, and the observed gene upregulation may induce natural competence within the airways. Following this rationale, we assessed natural competence *in vivo* by intranasal co-administration of a mixed bacterial inoculum containing heat-killed NTHi375 wild-type (WT) and live Δ*opsX* bacteria, a mutant previously shown to be attenuated (33), aiming to recover WT infection levels. However, the virulence of Δ*opsX* was not restored. Also, using already available H. influenzae RdKW20 derivative strains with a high natural transformation frequency, we co-administered a mix containing two RdKW20 strains with two different antibiotic resistance markers (Strep^R^ and Nov^R^) (34, 35), but did not recover double-positive Strep^R^Nov^R^ colonies after infection (data not shown). In summary, under the conditions tested, *in vivo* selectable DNA uptake, translocation, and homologous recombination was not observed.

### Correlation between bacterial gene upregulation and virulence.

**FIG 5:**
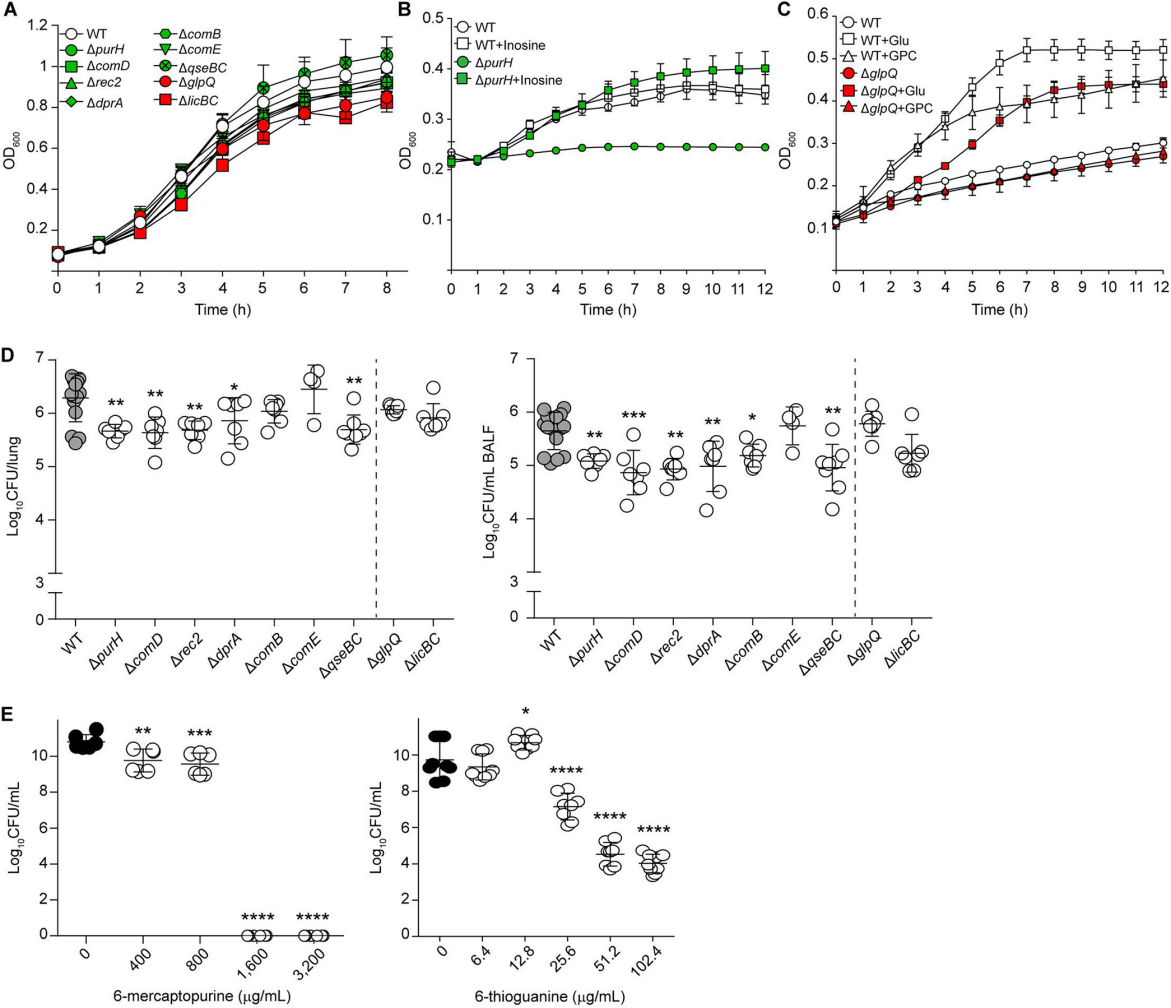
Antimicrobial effects of inactivating H. influenzae pathways differentially expressed upon airway infection. (A) Growth of NTHi375 WT, Δ*purH*, Δ*comD*, Δ*rec2*, Δ*dprA*, Δ*comB*, Δ*comE*, Δ*qseBC*, Δ*glpQ*, and Δ*licBC* strains in sBHI. Strains were grown in sterile 250-mL flasks with 25 mL sBHI and shaking; OD_600_ was recorded every hour for 8 h (mean ± SD). (B) Growth of NTHi375 WT and Δ*purH* strains in MM, in the absence or presence of 6.34 mM inosine. Strains were grown in 96-well plates, OD_600_ was recorded every hour for 12 h (mean ± SD). NTHi375Δ*purH* showed no growth in the absence of inosine (from 3 to 12 h, *P* < 0.05). NTHi375Δ*purH* growth was restored when inosine was added to the MM. (C) Growth of NTHi375 WT and Δ*glpQ* strains in CDM, in the absence or presence of 10 mM glucose or 10 mM GPC. Strains were grown in 96-well plates, OD_600_ was recorded every hour for 12 h (mean ± SD). The WT strain did not grow in the absence of glucose (from 2 to 12 h, *P* < 0.05) or GPC (from 2 to 12 h, *P* < 0.001). WT growth in the presence of GPC was lower than that in the presence of glucose (from 5 to 12 h, *P* < 0.01). NTHi375Δ*glpQ* only grew when glucose was added (from 3 to 12 h, *P* < 0.001). (D) CD1 mice were intranasally infected with NTHi375 WT or mutant strains (1 × 10^8^ CFU per mouse). Bacterial counts were determined at 12 hpi in lung (log_10_ CFU/lung) and BALF (log_10_ CFU/mL BALF) samples. Results are shown as mean ± SD. A significant reduction in bacterial load was observed upon infection with *purH*, *comD*, *rec2*, *dprA*, *comB*, and *qseBC* strains compared to the WT. Statistical comparisons of means were performed by one-way analysis of variance (ANOVA) and Dunnett’s multiple-comparison test (*, *P* < 0.05; **, *P* < 0.005; ***, *P* < 0.0001). (E) Purine analogs have an antimicrobial effect on H. influenzae growth. Determination of 6-mercaptopurine and 6-thioguanine inhibitory effects on NTHi375 strain upon growth in sBHI medium. Susceptibility to both molecules was dose-dependent. Results are shown as log_10_CFU/mL (mean ± SD). For each drug, statistical comparisons of means were performed by one-way ANOVA and Dunnett’s multiple-comparison test (*, *P* < 0.05; **, *P* < 0.01; ***, *P* < 0.001; ****, *P* < 0.0001). Reduced bacterial viability was observed at 400 μg/mL or higher concentrations of 6-mercaptopurine, and at 25.6 μg/mL or higher 6-thioguanine concentrations.

To gain insights into the importance of the observed differential gene expression in H. influenzae *in vivo* fitness, we next selected genes involved in purine biosynthesis (*purH*), iron uptake-sensing (*qseBC*/*firRS*) and natural competence (*comD*, *rec2*, *dprA*, *comB*, *comE*) whose expression was upregulated *in vivo*, and genes involved in LOS decoration (*glpQ* and *licBC*), which were downregulated *in vivo*, and generated mutants for each gene. None of these mutants presented growth defects in sBHI (Fig. 5A). As expected, Δ*purH* showed purine auxotrophy, restored by inosine addition (Fig. 5B); Δ*glpQ* was unable to grow using GPC as a carbon source (Fig. 5C) (36); and the Δ*comD*, Δ*rec2*, Δ*dprA*, Δ*comB*, and Δ*comE* mutants showed decreased transformation frequency compared to the WT strain (Table S3). To determine whether these genes play a role in bacterial fitness during infection, mice groups were independently infected with each mutant and processed at 12 hpi. Significantly lower counts were observed in both lung and BALF samples upon infection with NTHi375Δ*purH*, Δ*comD*, Δ*rec2*, Δ*dprA*, and Δ*qseBC* mutants than with the WT strain; also, lower counts were observed in BALF samples upon infection with the Δ*comB* mutant than in infection with the WT strain. In contrast, inactivation of the *in vivo* downregulated *licBC* or *glpQ* genes did not have significant effects on lung and BALF CFU counts (Fig. 5D).

Together, the correlations between upregulated bacterial gene expression during airway infection and gene contribution to infection suggest that *in vivo* RNA-seq may be useful when searching bacterial genes required for H. influenzae fitness within the airways.

### Purine analogs 6-thioguanine and 6-mercaptopurine inhibit *H. influenzae* growth.

Purine analogs are important therapeutics for acute leukemias and inflammatory bowel disease. Moreover, purine thio-derivatives have potential application as antimicrobials (37, 38). Here, we examined the effect of two purine analogs, 6-thioguanine and 6-mercaptopurine, on the growth of NTHi375 *in vitro*. We observed a significant dose-dependent reduction in bacterial viability after incubation. The extent of these inhibitory effects was heterogeneous between drugs. Significant bacterial viability loss was observed when using 400 μg/mL mercaptopurine, with complete loss at up to 1,600 μg/mL. This inhibitory effect was not reversible (data not shown). In contrast, significant bacterial viability loss was observed when using 25.6 μg/mL 6-thioguanine, and we did not reach a complete inhibition in the dose range tested (Fig. 5E).

In summary, the viability of H. influenzae 375 decreased when it was exposed to purine analogs in a dose-dependent manner, with varying efficacy between drugs. The susceptibility of H. influenzae to 6-thioguanine and 6-mercaptopurine suggests that *in vivo* gene expression profiling is a robust approach to identifying drug targets.

## DISCUSSION

In this study, we used *in vivo* RNA-seq to investigate the physiology and fitness of H. influenzae during murine lung infection, using a previously used model of acute infection and clearance. We observed upregulation of *de novo* purine biosynthesis which, in turn, contributed to H. influenzae airway infection. A link between bacterial purine synthesis and virulence has emerged, as different species require *de novo* nucleotide biosynthesis for full virulence (39). Our data show that this is also the case for H. influenzae. Moreover, bacteria salvage nucleotides from the environment to supplement *de novo* synthesis. Indeed, when it is a guanine auxotroph, H. influenzae may rely on guanine availability in the basal medium to support growth upon migration across the human epithelia (40). Here, besides upregulation of purine synthesis, we also observed upregulation of the guanine/adenine transporter HI0125 and of part of the natural transformation machinery in BALF samples.

Naturally competent bacteria pull DNA fragments from their environment into their cells. The most immediate consequence of DNA uptake is nutritional because DNA is a source of deoxyribonucleotides needed for replication of the cell’s own genome, and *de novo* nucleotide synthesis is costly in terms of energy and molecular constituents. Moreover, uptake of intact DNA is efficient at obtaining nucleotides because it limits losses due to diffusion and avoids the need for nucleoside rephosphosphorylation after uptake (30). Upregulation of a significant part of the natural transformation machinery in BALF samples, together with previously traced recombination events (41) and evidence for *in vivo* natural transformation by other airway bacteria (42), led us to try monitoring H. influenzae DNA uptake and recombination *in vivo*. We cannot exclude the limitations of our experimental design or the narrow time window for *in vivo* competence, but current evidence leads us to hypothesize that H. influenzae uptake of nucleobases or DNA may mostly have nutritional purposes. If so, restriction of these nutrients in the host niche could control infection, and nucleoside analogs or repurposing nucleotide synthesis inhibitors may be used in addition to standard antimicrobials (39). Supporting this notion, we show a H. influenzae growth-inhibitory effect for two purine analogs, 6-thioguanine and 6-mercaptopurine. These molecules utilize the same pathways as their natural counterparts, competing for both uptake and metabolism, and their misincorporation into nucleic acids leads to cytotoxicity (37, 38, 43). Although the targets of 6-thioguanine and 6-mercaptopurine are unknown in H. influenzae, growth inhibition was comparable on WT and Δ*purH* strains, suggesting PurH downstream effects (data not shown). For example, 6-thioguanine inhibits the Mycoplasma pneumoniae hypoxanthine guanine phosphoribosyl transferase (43). Moreover, the data presented in this work are from the laboratory culture of an individual H. influenzae strain. Bacteria were grown in sBHI medium, which differs from conditions encountered in the infecting niche and may modify the drug concentrations required to inhibit bacterial growth during infection. Despite their potential clinical implications, direct examination of the effects of these drugs on bacterial growth in more physiological conditions will provide information regarding their effects on bacterial dynamics during airway infection and their potential use at safe dose ranges. Bacterial targets, purine analog efficacy against H. influenzae clinical isolate collections, effects of exposure in terms of resistance development, and synergisms with other antimicrobial drugs, will be studied in future work.

We also observed *in vivo* downregulation of genes involved in LOS decoration. The PCho^–^ phenotype correlates with invasive infection by evasion of serum-mediated killing by C-reactive protein (CRP) (44, 45), and H. influenzae’s ability to reversibly inactivate *lic2A* by phase-variation may influence survival in niches where UDP-galactose levels are limiting (46). Current data suggest that the PCho^–^ and *lic2A*^–^ phenotypes may also be selected during lung infection. However, downregulated expression of genes involved in bacterial cell wall synthesis may be a more general theme, as observed for fatty acid, lipid A, and peptidoglycan synthesis pathways. Changes in Klebsiella pneumoniae lipid A structure and P. aeruginosa peptidoglycan composition have been reported, primarily influenced by environment conditions (47, 48). The consequences of the observed gene expression downregulation *in vivo* are currently unknown and will be assessed in future studies.

It should also be mentioned that our attempt to use ASM as *in vitro* approach to mimic *in vivo* conditions did not render the expected results. ASM was originally designed to model cystic fibrosis mucus (19), which may significantly differ from the murine airway environment used in this study to model H. influenzae respiratory infection. Also, ASM formulation needs further supplementation to allow H. influenzae growth, which may alter its mucus-mimicking purpose. Although it was not useful in this case, we contribute to expanding the range of *in vitro*-defined media suitable for H. influenzae growth. Another related aspect worth mentioning is the murine lung infection model used in this work. We initially considered using our murine emphysematous lung infection model (49), which may be more relevant to human lung infection in persons with COPD. However, our methodology led us to use BALF samples to obtain RNA in sufficient quantities to study the pathogen transcriptome, and we could not use murine emphysematous lung infection because BALF sampling in this model has been previously shown to be challenging and poorly reproducible (49). While the data generated from the *in vivo* transcriptome of the lung during H. influenzae infection only allowed us to study the host transcriptome, we speculate that adequate sequence coverage could be obtained by increasing the sequencing depth to obtain adequate coverage for statistical analysis of bacterial gene expression in the lung. These aspects will be considered in further studies.

Regarding the host, dysregulation of the cytoskeleton and pro-inflammatory responses was observed in infected lung samples, aligned with previous profiling of the transcriptome of airway-cultured cells upon H. influenzae infection (14). We also observed differential gene expression compatible with ECM remodeling upon infection. Genes encoding fibrillin (FBN1 and FBN2), hemicentin 1 (HMCN1), laminin (LAMB1, LAMB2, and LAMB3), several collagen proteins, ADAMTSL1, 2, and 3, and the matrix metalloproteinase (MMP) MMP15 were downregulated; genes encoding MMP3, MMP8, MMP9, and MMP25 were upregulated, aligned with previous observations (14).

**FIG 6:**
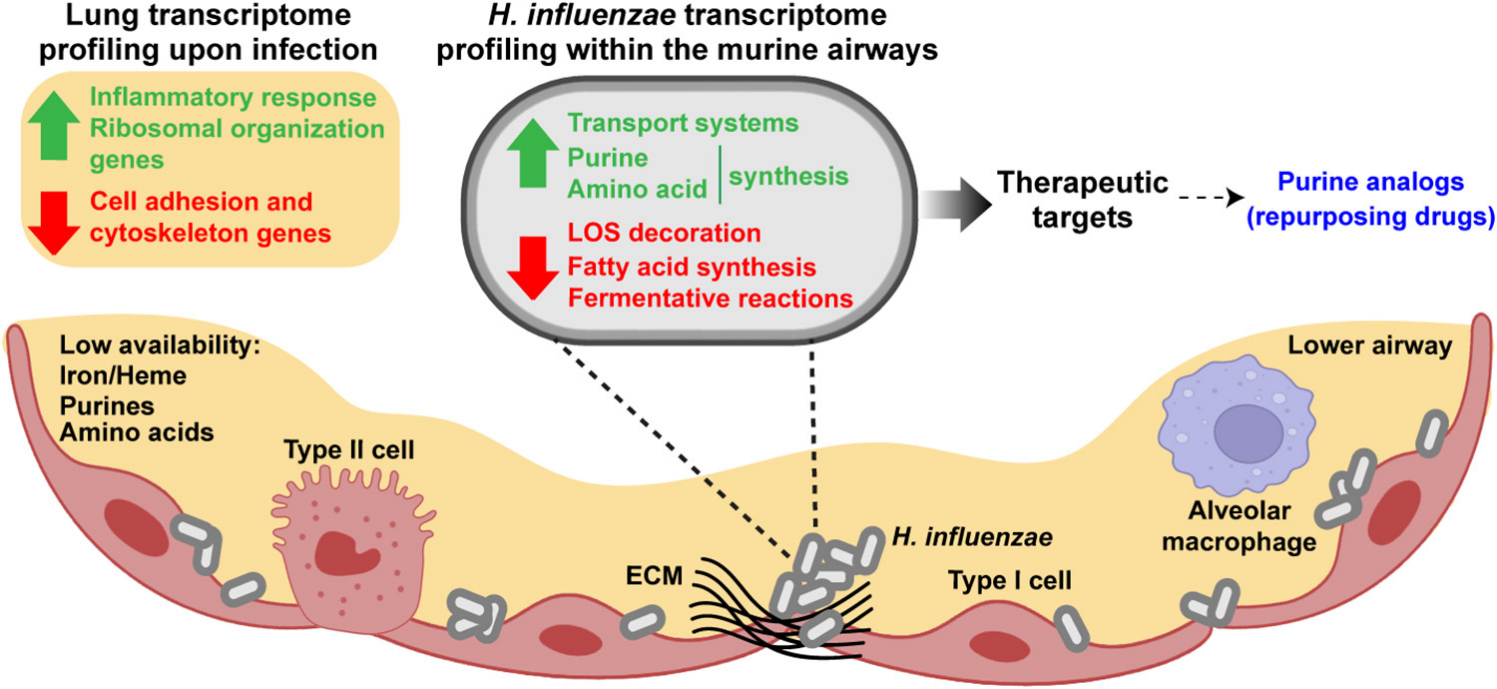
Schematic representation of H. influenzae-lower airway interplay, where novel features were uncovered by *in vivo* transcriptomic profiling. Expression of murine host genes associated with inflammatory responses and ribosomal organization was shown to be upregulated during infection; cell adhesion and cytoskeleton genes were downregulated. Bacterial metabolic requirements during infection may include, among others, purine and amino acid biosynthesis, and a whole range of transport systems (iron/heme, nucleobases, ions, amino acids, etc.) which may contribute to overcoming nutritional limitations within the infected niche. Conversely, modulation of LOS structure, likely by lowering PCho, sialic acid, and digalactose decoration, together with lower expression of genes involved in fatty acid synthesis and fermentative reactions, was observed. Upregulated genes/pathways are potential therapeutic targets, as suggested by the inhibitory effect of purine analogs on H. influenzae growth. Green, gene groups (host or bacterial) upregulated upon infection; red, gene groups (host or bacterial) downregulated upon infection.

Overall, we present novel genome-wide information on the H. influenzae-host airway cross-talk (summarized in Fig. 6). Our results add, from a different perspective, to those obtained by genome-wide screening of H. influenzae genes required in the murine lower airway where, among others, purine metabolism genes (*purM*, *purE*, *purH*, *purD*, *purL*, *purC*, *purF*, *guaB*, *purB*) were also shown to contribute to this bacterial fitness *in vivo* (6). Here, we mainly focused on bacterial metabolism because a significant number of metabolic genes were differentially expressed during infection, and H. influenzae metabolism during infection is poorly understood. Moreover, modulation of the host metabolic environment has shown promise for treating other diseases characterized by fast-growing and invasive cells, such as cancer (37), and has immediate therapeutic potential for treating infections. Our data also suggest that altering the pathogen’s metabolism may impair its fitness, and therapeutic interference with bacterial purine biosynthesis deserves further exploration. Full understanding of pathogen metabolic rewiring upon infection, together with systematic metabolic evaluation of individual strains by computational means, will be key to designing more precise and accurate treatments.

## MATERIALS AND METHODS

### Bacterial strains and growth conditions.

The strains used in this study are listed in Table S1. Generation of H. influenzae mutant strains is described in the supplemental material (see Supplementary Methods in Supplemental File 3). H. influenzae strains were grown at 37°C, 5% CO_2_ on PolyViteX (PVX) agar (bioMérieux); BHI agar (Condalab, 1400.10; Oxoid, CM1135) supplemented with 10 μg/mL hemin (Merck, H9039) and 10 μg/mL NAD (NAD, Merck, N0632), referred to as sBHI agar; or supplemented Haemophilus Test Medium agar (HTM, Oxoid, CM0898), referred to as sHTM agar. NTHi liquid cultures were grown at 37°C, 5% CO_2_ in the following media: (i) sBHI; (ii) ASM, freshly prepared exactly as described by Kirchner et al. (19) and supplemented to allow H. influenzae growth by adding 85 μg/mL uracil, 1.68 mg/mL inosine, 20 mM glucose, 10 μg/mL hemin, and 10 μg/mL NAD, referred to as sASM; (iii) chemically defined medium (CDM) (18, 50, 51); and (iv) minimal medium (MM) (17). Erythromycin at 11 μg/mL (Erm_11_), 50 or 150 μg/mL spectinomycin (Spec_50_; Spec_150_), 2.5 μg/mL novobiocin (Nov_2.5_), or 100 μg/mL streptomycin (Strep_100_) were used when required. Escherichia coli was grown on Luria Bertani (LB) or LB agar at 37°C, with 100 μg/mL ampicillin (Amp_100_), 150 μg/mL Erm (Erm_150_), or 50 μg/mL Spec (Spec_50_) when necessary.

### Bacterial growth.

NTHi strains were grown on PVX agar for 12 h. Subsequently, and depending on the assay, growth was monitored as follows. (i) Two to five colonies were inoculated in 10 mL sBHI and incubated for 11 h with shaking (100 rpm). Cultures were then diluted to an optical density at 600 nm (OD_600_) of 0.07 in sBHI, incubated in sterile 250-mL flasks with 25 mL sBHI and shaking (200 rpm). OD_600_ was recorded every 1 h for up to 8 h. (ii) Two to five colonies were inoculated in 20 mL sBHI and incubated as described above. Cultures were then diluted to OD_600_ = 0.1 in sASM, incubated in sterile 250-mL flasks with 25 mL sASM and shaking (200 rpm). OD_600_ was recorded as described above. All flask experiments were performed on at least three independent occasions (*n* ≥ 3). (iii) Bacterial suspensions collected from PVX agar overnight (o/n) growth were normalized to OD_600_ = 1 in CDM, and 40-μL aliquots were transferred to individual wells in 96-well plates (Sarstedt) with 160 μL CDM; when indicated, CDM was supplemented with 10 mM glucose or 10 mM l-α-glycerophosphocholine (Merck, G5291). GPC stock solutions were prepared in distilled water at 190 mM. Plates were incubated at 37°C for 12 h in a Spectro Star Nano (BGM Labtech). (iv) Bacterial suspensions collected from PVX agar o/n growth were normalized to OD_600_ = 1 in MM, and 40-μL aliquots transferred to individual wells in 96-well microtiter plates with 160 μL MM, in the absence or presence of 6.34 mM inosine. Plates were incubated at 37°C for 12 h in a Spectra Max 340 PC (Molecular Devices). In (iii) and (iv), OD_600_ was determined every hour, and growth curves were corrected to their respective blank values (CDM or MM). All 96-well experiments were performed in triplicate on at least three independent occasions (*n* ≥ 3).

### Antibacterial effect of purine analogs.

6-Thioguanine (Merck, A4882) and 6-mercaptopurine monohydrate (Merck, 852678) were used by freshly preparing 25 and 100 mg/mL stock solutions of each molecule in 1 M NaOH, respectively, filtered and used at the indicated concentrations. Purine analog susceptibility was determined as follows: a stock solution of 25 mg/mL 6-thioguanine was diluted to 6.56 mg/mL in sBHI, and a stock solution of 100 mg/mL 6-mercaptopurine was diluted to 25.6 mg/mL in sBHI. Next, 200 μL of 6.56 mg/mL 6-thioguanine or 200 μL of 25.6 mg/mL 6-mercaptopurine were added to individual wells in column 1 in 96-well plates (Sarstedt). Then, 100-μL aliquots of sBHI were transferred to individual wells in the rest of the plate. Next, 100-μL aliquots were serially transferred from wells in column 1 to wells in column 2 and up to column 11, where 100 μL was discarded. A suspension of PVX agar with freshly grown bacteria was generated in sBHI, adjusted to 0.5 MacFarland (OD_600_ = 0.063) and diluted 1:100. Next, 100-μL bacterial aliquots were transferred to each well. Plates were incubated for 24 h at 37°C, 5% CO_2_, without shaking. Bacterial growth controls (without drugs) were included in each case (column 12). Culture samples were serially diluted and plated on sHTM agar. Data are shown as log CFU/mL; experiments were performed in triplicate on three independent occasions (*n* = 3).

### Animal handling.

CD1 female mice (18 to 20 g) aged 4 to 5 weeks (Charles River Laboratories) were housed under pathogen-free conditions at the Instituto de Agrobiotecnología, Consejo Superior de Investigaciones Científicas (IDAB-CSIC) animal facility (registration no. ES/31-2016-000002-CR-SU-US). Animal handling and procedures were in accordance with European (Directive 2010/63/EU) and national (RD53/2013 and RD118/2021) legislation, with authorization from the Universidad Pública de Navarra (UPNA) and CSIC Animal Experimentation Committees and the local government (Protocol no. PI007/19).

### Animal procedures.

For mouse lung infection, NTHi cultures were exponentially grown (OD_600_ = 0.3) in sBHI and preserved at 80°C until use. For intranasal infection, bacteria-containing cryotubes were thawed and centrifuged, and the pellets were resuspended in phosphate-buffered saline (PBS) to obtain ~5 × 10^9^ CFU/mL. Then, 20 μL of the bacterial suspension was placed at the opening of the nostrils until complete inhalation by each mouse (~1 × 10^8^ CFU/mouse), which was previously anesthetized with ketamine (Imalgene, Merial) and xylazine (Rompun, Bayer AG) (3:1). When indicated, mice were euthanized by cervical dislocation and lungs were aseptically removed, weighed in sterile bags (Stomacher80, Seward Medical), and homogenized 1:10 (wt/vol) in PBS. Each homogenate was serially 10-fold diluted in PBS and plated in triplicate on sHTM agar to determine the number of viable bacteria (CFU counts). When specified, the left lung was processed for CFU counting and the right lung for RNA extraction. When needed, BALF samples were obtained and processed.

### NTHi infection for *in vivo* RNA-seq.

NTHi375 WT was grown on PVX agar for 12 h; 2 to 5 colonies were inoculated in 20 mL sBHI and incubated for 11 h with shaking (100 rpm). Cultures were diluted to OD_600_ = 0.07 in sBHI and grown in triplicate in sterile 250-mL flasks with 25 mL sBHI and shaking (200 rpm) to OD_600_ = 0.3 prior to collection. Approximately 5 × 10^9^ CFU of these cultures were used for each separate RNA extraction (see below). Two murine infection assays were performed. In the first one, to generate material for murine lung RNA-seq, 12 CD1 animals were divided into two groups, non-infected (*n* = 6) and infected (*n* = 6), and each group was divided into three subgroups (*n* = 2). Mice in each subgroup of the infected group were administered bacteria from one of the three collected bacterial suspensions at 20 μL (~1 × 10^8^ CFU/mouse). Mice in each subgroup were sampled at 12 hpi and underwent right lung processing for RNA extraction. The left lungs of the infected group were processed for CFU counts. In the second assay, to generate material for bacterial RNA-seq, 84 CD1 mice were divided into three groups (*n* = 28) and infected with 20 μL (~1 × 10^8^ CFU/mouse) of each of the three collected WT bacterial suspensions. The three groups were sequentially infected on the same day with the three independently generated bacterial cultures (see above; technical replicates). Mice were sampled at 12 hpi and BALF samples were obtained by injecting 3 mL PBS into the lung. BALF samples corresponding to each group were pooled, filtered using a 5.0-μm syringe filter, and used for serial dilution plating to quantify viable bacteria and for RNA extraction.

### *H. influenzae* infection for *in vivo* screening validation.

Single-bacterial strain infections were performed. For this purpose, 70 CD1 mice were divided into 10 groups (*n* = 7) and infected with bacterial suspensions prepared as described above (“NTHi infection for *in vivo* RNA-seq”) at ~1 × 10^8^ CFU/mouse. At 12 hpi, mice were euthanized and BALF and lung samples were obtained and processed for CFU counts. BALF samples were obtained by perfusion and collection of 0.7 mL PBS using a sterile 20-G VialonTM intravenous catheter inserted into the trachea, serially diluted in PBS, and plated in triplicate on sHTM agar. By following standardized procedures (52), we considered that we could have a minimum of 3.3 CFU in a 1-mL sample without detecting bacteria (limit of detection < 3 to 4 CFU/mL BALF), rendering log_10_ = 0.52.

### NTHi infection for assessing natural transformation.

Two types of assay were performed. In the first one, mice (*n* = 7) were intranasally co-infected (10:1) with NTHi375 WT strain heat-killed (HK) and the previously shown attenuated mutant NTHi375 Δ*opsX* (33), and, at 12 hpi, processed for lung homogenate CFU counting to assess reversion of the attenuation. In the second one, four CD1 mice were intranasally co-infected (1:1) with two RdKW20 derivative strains carrying different antibiotic resistance markers (RdKW20-Str^R^, P193; RdKW20-Nov^R^, P194) (34), 1 × 10^8^ CFU/mouse. At 12 hpi, BALF samples were obtained by a 3-mL PBS injection into the lung and were further collected, pooled, filtered using a 5.0-μm syringe filter, pelleted by centrifugation at 14,000 rpm for 10 min, and resuspended in 3 mL PBS for plating of the entire volume on sHTM + Strep_100_ + Nov_2,5_ agar.

### RNA extraction, purification, and further processing.

RNA for sequencing was isolated from bacteria grown in sBHI or in sASM to OD_600_ = 0.3, or from murine BALF and lung samples. Methodological details are provided in the Supplementary methods, see Supplemental File 3. The presence and quantity of eukaryotic and bacterial genes in RNA samples was tested by RT-qPCR analysis. All samples were treated with Ribo-Zero Gold Epidemiology (Illumina) for rRNA depletion, and RNA integrity was reassessed. rRNA-depleted mRNA samples were fragmented and prepared using Illumina TruSeq RNA SMARTer Strander V3 library preparation. Libraries were checked for quality control with Bioanalyzer (Agilent) and sequenced on an Illumina HiSeq platform with 2 × 150-bp reads by Admera Health. Sample types: (i) NTHi grown in sBHI (OD_600_ = 0.3) or sASM (OD_600_ = 0.3); (ii) BALF samples from infected mice; (iii) lung samples from infected mice; and (iv) non-infected murine lung samples. Sixty million 2 × 150-bp reads were dedicated for the three replicates of each sample type.

### RNA-seq data analysis.

RNA-seq reads were analyzed with CLC Genomics workbench software version 21.0.5 (Qiagen) by using the H. influenzae NTHi375 (CP009610.1) and Mus musculus (GRCm38 – mm10) genomes as references. RNA reads were trimmed to remove read-through adapter sequencing, low-quality sequences (limit = 0.05), ambiguous nucleotides (max = 2), and homopolymer G sequences on the 3′ end. Reads were then mapped in pairs against the gene regions of the reference genomes (annotation version 108.20200622) using the following settings: mismatch cost = 2, insertion cost = 3, deletion cost = 3, length fraction = 0.8, similarity fraction = 0.8. Reads per kilobase per million (RPKM) were calculated using the default parameters in CLC Genomics to determine relative changes in gene expression (*P* < 0.05). Fold changes in gene expression and statistical analyses were performed using an EDGE test with Bonferroni correction. NTHi differentially expressed genes, pathways, or biological functions with fold change >1.5, were searched in KEGG, PubMed, and UniProt; in addition, BLASTn was performed using the sequence of each differentially expressed bacterial gene as a query against all H. influenzae genomes available in NCBI. The remaining unidentified genes were grouped as hypothetical. For functional analysis of host transcriptome, only genes with Bonferroni-corrected *P* < 0.05 and fold change >2 were used. rRNA genes were discarded during the analysis. GO Term biological processes analysis was performed using WebGestalt online tool (2019 version) using default parameters (53). Affinity propagation was used to cluster similar gene sets to reduce redundancy in the report using default analysis parameters. STRING analyses of differentially regulated genes were performed and visualized by using Cytoscape version 3.7.1 (54, 55). Mean RPKM values of differentially regulated genes from each sample in each experimental group were used to perform principal-component analysis using the ClustVis web tool with default parameters (56).

### Statistical analyses.

In all cases, *P* < 0.05 was considered statistically significant. Analyses were performed using the Prism software version 7 for Mac (GraphPad Software) statistical package and are detailed in each figure legend.

### Data availability.

RNA-seq raw sequencing data reads have been deposited in the NCBI Sequence Read Archive (SRA) and are available under BioProject no. PRJNA859909.
