# Supplementary material for: In Vivo Genome-Wide Gene Expression Profiling Reveals That Haemophilus influenzae Purine Synthesis Pathway Benefits Its Infectivity within the Airways
Source: Microbiol Spectr. 2023 May 17;11(3):e00823-23. doi: 10.1128/spectrum.00823-23 (PMC10269889; doi:10.1128/spectrum.00823-23)
Supplement: Supplemental file 3 — Tables S1-S3; Figures S1-S3. Download spectrum.00823-23-s0001.docx, DOCX file, 1.1 MB [file spectrum.00823-23-s0001.docx]

**Supplemental Material**

***In vivo* genome-wide gene expression profiling reveals that *Haemophilus influenzae* purine synthesis pathway benefits its infectivity within the airways**

Begoña Euba^a^, Celia Gil-Campillo^a,b^, Javier Asensio-López^a,c^, Nahikari López-López^a,b^, Emel Sen-Kilic^d,e^, Roberto Díez-Martínez^f^, Saioa Burgui^c^, Mariette Barbier^d,e#^, Junkal Garmendia^a,b,g#^

^a^Instituto de Agrobiotecnología, Consejo Superior de Investigaciones Científicas (IdAB-CSIC)-Gobierno de Navarra, Mutilva, Spain; ^b^Centro de Investigación Biomédica en Red de Enfermedades Respiratorias (CIBERES), Madrid, Spain; ^c^Asociación de la Industria Navarra (AIN)-Gobierno de Navarra, Cordovilla, Spain; ^d^Vaccine Development Center, West Virginia University Health Sciences Center, Morgantown, West Virginia, USA; ^e^Department of Microbiology, Immunology and Cell Biology, West Virginia University School of Medicine, Morgantown, West Virginia, USA; ^f^Telum Therapeutics, Noain, Spain; ^g^Conexión Nanomedicina-CSIC, Madrid, Spain

**Content:**

**Dataset S1.** Murine genes differentially expressed upon airway infection by *H. influenzae* NTHi375.

**Dataset S2.** Bacterial genes differentiallly expressed (up- or down-regulated) upon: (i) murine infection (NTHi sBHI compared to BALF samples), sheets 1 and 2; (ii) NTHi sBHI compared to sASM samples, sheets 3 and 4; (iii) NTHi sASM compared to BALF samples, sheets 5 and 6.

**Supplementary methods.**

**Table S1.** Bacterial strains and plasmids used in this study.

**Table S2.** Primers used in this study.

**Table S3.** Transformation frequency of NTHi375 WT and mutant strains, in MIV medium, by using MAP-7 gDNA.

**Figure S1.** *H. influenzae* differentially expressed genes involved in amino acid synthesis (NTHi sBHI *vs* BALF samples).

**Figure S2.** Growth of *H. influenzae* NTHi375 in sASM.

**Figure S3.** STRING analyses of *H. influenzae* genes up- (upper panel) or down-regulated (lower panel) when comparing sBHI to sASM samples.

**Supplementary methods.**

**Generation of *H. influenzae* mutant strains.** Plasmids and primers are shown in **Tables S1** and **S2**, respectively. For generation of *H. influenzae* mutants, a DNA fragment containing each gene/operon and its respective flanking regions was PCR amplified with Phusion polymerase (Thermo Scientific) using NTHi375 genomic DNA as template and primers gene+flanking region-F1 and gene+flanking region-R1, and cloned into pJET1.2/blunt (Thermo Scientific), generating a collection of pJET1.2-*gene* plasmids. Three gene disruption strategies were employed: (i) an *ermC*-based disruption strategy: in each case, the cloned PCR product was disrupted by inverse PCR with Phusion polymerase, using primers *gene*-F2 and *gene*-R2. An internal fragment was replaced by a blunt-ended *ermC* resistance cassette excised by *Sma*I digestion from pBSLerm (1), generating the respective collection of pJET1.2-*gene*::*ermC* plasmids, used as a template to amplify each *gene*::*ermC* disruption cassettes with primers gene+flanking region-F1 and gene+flanking region-R1; (ii) *spec*-based disruption strategy A: in each case, the cloned PCR product was disrupted by inverse PCR with Phusion polymerase using primers *gene*-F2 and *gene*-R2. An internal fragment was replaced by a blunt-ended Spec resistance cassette excised by *EcoR*V digestion from pRSM2832, generating the respective collection of pJET1.2-*gene*::*spec* plasmids, used as a template to amplify each *gene*::*spec* disruption cassettes with primers gene+flanking region-F1 and gene+flanking region-R1; (iii) Spec-based disruption strategy B: for each gene, a Spec resistance gene was independently PCR amplified from pRSM2832 using gene-specific mutagenic primers *gene*-F2 and *gene*-R2. *E. coli* SW102 cells were prepared for recombineering, co-electroporated with pJET1.2-*gene* (Amp^r^) (50 ng), and the *gene*-specific mutagenic cassette (Spec^r^) (200 ng) (2). Mutagenized clones containing pJET1.2-*gene::spec* were selected on LB agar with Amp_100_ and Spec_50_. This plasmid was used as a template to amplify the *gene::spec* disruption cassette with primers *gene*-F1 and *gene*-R1. In all cases, disruption cassettes were independently used to transform *H. influenzae* strains by using the MIV method (3). Transformants were selected on sHTM agar with Erm_11_ to obtain NTHi375Δ*purH*, NTHi375Δ*comD*, NTHi375Δ*rec2*, NTHi375Δ*dprA*, NTHi375Δ*comB*, NTHi375Δ*comE*, NTHi375Δ*qseBC* mutants. Transformants were selected on sHTM agar with Spec_50_ to obtain NTHi375Δ*glpQ*. In all cases, mutations were confirmed by PCR.

**RNA extraction, purification and further processing.** RNA for sequencing was isolated from two different sample types. First, NTHi was grown for 12 h on PVX chocolate agar. Two to five colonies were inoculated into 20 mL sBHI and grown for 12 h at 100 r.p.m. Cultures were then diluted into 20 mL fresh sBHI to OD_600_=0.07 or into 20 mL fresh sASM to OD_600_=0.1, and grown to OD_600_=0.3 at 200 r.p.m. Bacterial RNA was isolated using NucleoSpin RNA kit (Macherey-Nagel) as specified by the instructions of the manufacturer. Briefly, ∼5x10^9^ CFU were pelleted by centrifugation at 14,000 r.p.m. for 5 min, resuspended in 100 µL TE buffer (10 mM Tris-HCl, 1 mM EDTA, pH8) containing 1 mg/mL lysozyme by vigorous vortexing, and incubated at 37ºC for 10 min. Cells were lysed with 350 μL Buffer RA1 and 3.5 μL β-mercaptoethanol. Lysates were filtered through NucleoSpin Filter units to reduce viscosity, and mixed with 350 μL of 70% ethanol. RNA was applied to NucleoSpin RNA columns, salt was removed using membrane desalting buffer (MDB), one on-column rDNase treatment step was included, samples were cleaned with RAW2 and RA3 buffers, and RNA was eluted. Secondly, BALF and lung samples were processed using the same kit with the following modifications: (i) for BALF, samples were filtered using a 5.0 µm syringe filter before pelleted; (ii) for lungs, fresh lung tissue up to 30 mg was disrupted in 600 μL Buffer RA1 with 6 mL β-mercaptoethanol, and homogenized using a conventional rotor-stator homogenizer (IKA Ultra-turrax T10 basic homogenizer and dispersing element S10N-5G) prior RNA extraction. To prevent any DNA interference, a second DNase digestion was performed in RNA solutions using RNase-free DNase (Qiagen) and cleaned on another RNeasy Mini column (Qiagen).

RNA for relative quantification by RT-qPCR was isolated as it follows: NTHi strains were grown in sBHI up to OD_600_=0.3, or were grown in sBHI up to OD_600_=0.3 and then transferred to MIV for 100 min (depending on the assay). In all cases, 7 mL were recovered from each culture, pelleted (4,000 r.p.m, 4 min), flash frozen, and stored at -80ºC. Total RNA was isolated using TRIzol reagent (Invitrogen). BALF samples used for RT-qPCR are those indicated above.

In all cases, purified RNA was quantified on a Nanodrop One^C^ (Thermofisher Scientific), and total RNA integrity determined using RNA 6000 Nano LabChips (Agilent 2100 Bioanalyzer).

**RT-qPCR.** Reverse transcription was performed using 1 µg RNA by PrimerScript RT Reagent kit (Takara). cDNA diluted 1:10 was used as template in a 20 µL reaction mixture containing 1X SYBR Premix Ex Taq II (Tli RNaseH Plus) (Takara), and specific primers pairs for each gene (**Table S2**), designed with Primer3 software. Fluorescence was analyzed with AriaMx Real-Time PCR System (Agilent Technologies). The comparative threshold cycle (Ct) method was used to obtain relative quantities of mRNA that were normalized using bacteria *gyrA* or mouse *gapdh* genes as endogenous controls. Bacterial cultures were grown at least three times, BALF and lung samples were obtained in triplicate, and all samples were processed with technical triplicates (n≥3).

**Determination of *H. influenzae in vitro* natural transformation frequency.** Natural transformation by NTHi375 strains (WT and NTHi375Δ*comB*, NTHi375Δ*comD*, NTHi375Δ*comE*, NTHi375Δ*rec2*, NTHi375Δ*dprA* mutants) was determined by following the previousy described M-IV medium-based procedure (4), with slight modifications. Briefly, NTHi strains were grown in 10 mL sBHI for 11 h with shaking (100 r.p.m.). Cultures were then diluted to OD_600_=0.07 in sBHI, incubated in sterile 250 mL flasks with 17.5 mL of sBHI and shaking (200 r.p.m.), and grown to OD_600_=0.2-0.3. Cultures were then pelleted at room temperature (4 min, 6,000 x g), washed once with 24 mL of freshly prepared M-IV medium at room temperature, pelleted again, and resuspended in 25 mL M-IV. Cell suspensions were transferred to 250 mL sterile flasks and incubated with shaking (100 r.p.m.) for 100 min at 37ºC, 5% CO_2_. Then, we added ≥ 1 μg genomic DNA of the MAP7 (5) strain (P192) to 1 mL of each culture of competent cells, and mixed gently. Cells and DNA were incubated for 30 min at 37ºC with shaking (180 r.p.m.). Next, we added 2 mL of pre-warmed sBHI, and cultures were incubated for 30 min at 37ºC with shaking (180 r.p.m.), followed by serial dilution and plating on sHTM agar in the absence or presence of Nov_2,5_, to further calculate the transformation frequency of every strain being tested (nº CFU/mL grown on sHTM agar+Nov / nºCFU/ml grown on sHTM agar).

**Table S1.** Bacterial strains and plasmids used in this study.

| **Strain / Plasmid** | | **Description** | **Source** | | |
| --- | --- | --- | --- | --- | --- |
| **Strain** | |  |  | | |
| ***E. coli*** | |  |  | | |
| TOP10 | | Cloning strain. F- *mcrA* Δ(*mrr-hsd*RMS-*mcr*BC) Φ80*lac*ZΔM15 Δ*lac*X74 *rec*A1 *ara*D139 Δ(*ara,leu*)7697 *gal*U*gal*K*rps*L (Str^R^) *end*A1 *nup*G | Thermofisher Scientific | | |
| SW102 | | Derived from DY380; it contains a defective *λ* prophage with the recombination proteins *exo*, *bet*, and *gam* being controlled by the temperature-sensitive repressor *cI857* | (2) | | |
| ***H. influenzae*** | |  |  | | |
| NTHi375 | | Wild-type, otitis media clinical isolate | (6) | | |
| NTHi375Δ*purH*/P993 | | *purH::ermC*, Erm^R^ | This study | | |
| NTHi375Δ*comD*/P987 | | *comD::ermC*, Erm^R^ | This study | | |
| NTHi375Δ*rec2*/P984 | | *rec2::ermC*, Erm^R^ | This study | | |
| NTHi375Δ*dprA*/P985 | | *dprA::ermC*, Erm^R^ | This study | | |
| NTHi375Δ*comB*/P986 | | *comB::ermC*, Erm^R^ | This study | | |
| NTHi375Δ*comE/*P1166 | | *comE::ermC*, Erm^R^ | This study | | |
| NTHi375Δ*qseBC*/P1084 | | *qseBC::ermC*, Erm^R^ | This study | | |
| NTHi375Δ*glpQ*/P908 | | *glpQ::spec*, Spec^R^ | This study | | |
| NTHi375Δ*licBC*/1227 | | *licBC::ermC*, Erm^R^ | (7) | | |
| NTHi375Δ*opsX/*P350 | | *opsX::ermC*, Erm^R^ | (8) | | |
| P192 (MAP7) | | Rd Nov^R^Nal^R^Str^R^Spec^R^Kan^R^Rif^R^Erm^R^(aka RR666) | (5) | | |
| P193 | | Rd Str^R^, made as above but selecting for Str^R^(aka RR514) | (5) | | |
| **Plasmid** | |  |  | | |
| pJET1.2/blunt | | Cloning vector | Life Technologies | | |
| pBSLerm | Plasmid containing an Erm resistance cassette (Erm^R^) | | | (1) |  |
| pRSM2832 | pKD13 derivative carrying a cassette containing a Spec resistance gene flanked by FRT sites | | | (2) |  |
| pJET1.2-*purH* | pJET1.2 derivative containing a 3,523 bp DNA fragment carrying the *purH* gene (1,599 bp) and its upstream (924 bp) and downstream (1,000 bp) flanking regions | | | This study |  |
| pJET1.2-*purH*::*ermC* | pJET1.2-*purH* derivative containing a 3,358 bp DNA fragment carrying a *purH::ermC* disruption cassette | | | This study |  |
| pJET1.2-*comD* | pJET1.2 derivative containing a 2,414 bp DNA fragment carrying the *comD* gene (414 bp) and its upstream (1,000 bp) and downstream (1,000 bp) flanking regions | | | This study |  |
| pJET1.2-*comD*::*ermC* | pJET1.2-*comD* derivative containing a 3,354 bp DNA fragment carrying a *comD::ermC* disruption cassette | | | This study |  |
| pJET1.2-*rec2* | pJET1.2 derivative containing a 4,367 bp DNA fragment carrying the *rec2* gene (2,367 bp) and its upstream (1,000 bp) and downstream (1,000 bp) flanking regions | | | This study |  |
| pJET1.2-*rec2*::*ermC* | pJET1.2-*rec2* derivative containing a 3,768 bp DNA fragment carrying a *rec2::ermC* disruption cassette | | | This study |  |
| pJET1.2-*dprA* | pJET1.2 derivative containing a 3,122 bp DNA fragment carrying the *dprA* gene (1,122 bp) and its upstream (1,000 bp) and downstream (1,000 bp) flanking regions | | | This study |  |
| pJET1.2-*dprA*::*ermC* | pJET1.2-*dprA* derivative containing a 3,441 bp DNA fragment carrying a *dprA::ermC* disruption cassette | | | This study |  |
| pJET1.2-*comB* | pJET1.2 derivative containing a 2,266 bp DNA fragment carrying the *comB* gene (507 bp) and its upstream (859 bp) and downstream (900 bp) flanking regions | | | This study |  |
| pJET1.2-*comB*::*ermC* | pJET1.2-*comB* derivative containing a 3,130 bp DNA fragment carrying a *comB::ermC* disruption cassette | | | This study |  |
| pJET1.2-*comE* | pJET1.2 derivative containing a 3,247 bp DNA fragment carrying the *comE* gene (1,338 bp) and its upstream (1,000 bp) and downstream (909 bp) flanking regions | | | This study |  |
| pJET1.2-*comE::ermC* | pJET1.2-*comE* derivative containing a 3,541 bp DNA fragment carrying a *comE::ermC* disruption cassette | | | This study |  |
| pJET1.2-*qseBC* | pJET1.2 derivative containing a 4,009 bp DNA fragment carrying the *qseBC* gene (2,018 bp) and its upstream (991 bp) and downstream (1,000 bp) flanking regions | | | This study |  |
| pJET1.2-*qseBC*::*ermC* | pJET1.2-*qseBC* derivative containing a 3,350 bp DNA fragment carrying a *qseBC::ermC* disruption cassette | | | This study |  |
| pJET1.2-*glpQ* | pJET1.2 derivative containing a 3,094 bp DNA fragment carrying the *glpQ* gene (1,095 bp) and its upstream (1,000 bp) and downstream (999 bp) flanking regions | | | This study |  |
| pJET1.2-*glpQ::spec* | pJET1.2-*glpQ* derivative containing a 4,090 bp DNA fragment carrying a *glpQ::spec* disruption cassette | | | This study |  |

**Table S2.** Primers used in this study.

| **Primer name** | **Primer ID** | **Primer sequence (5’-3’)** | **Purpose** | **Source** |
| --- | --- | --- | --- | --- |
| HI0125-qPCR-F | 2039 | CGTGAGCGATATTGGATGGT | qRT-PCR | This study |
| HI0125-qPCR-R | 2040 | GCAGTATTCTTAATGGGGGTTG | qRT-PCR | This study |
| *tbpA*-qPCR-F3 | 1471 | AAGTAAGAGATCGTAAAGATAATGAAGTAACTG | qRT-PCR | (9) |
| *tbpA*-qPCR-R3 | 1472 | ACCGCGACCTTGTTCTACAACT | qRT-PCR | (9) |
| *purM*-qPCR-F | 2025 | TTGAGCCGAGTGCGATAAG | qRT-PCR | This study |
| *purM*-qPCR-R | 2026 | TGCCAGGAATGTATCACGAA | qRT-PCR | This study |
| *purE*-qPCR-F | 2035 | CATGTGGAAATCGTCTCTGCT | qRT-PCR | This study |
| *purE*-qPCR-R | 2036 | ATCATGCCGGGTAAATGTG | qRT-PCR | This study |
| *purH*-qPCR-F | 1835 | CTGCTGGAAAGTGGCAAAA | qRT-PCR | This study |
| *purH*-qPCR-R | 1836 | GGAATGGGAAGAACGCATC | qRT-PCR | This study |
| *purK*-qPCR-F | 2037 | GAAATTGAACGCTGGGAAAA | qRT-PCR | This study |
| *purK*-qPCR-R | 2038 | CACCAAGGCGAGGTAGAAAG | qRT-PCR | This study |
| *purN*-qPCR-F | 2027 | ACGGGTTTTAGCCTCCACTT | qRT-PCR | This study |
| *purN*-qPCR-R | 2028 | AGATGGTGGTGCGATTGTCT | qRT-PCR | This study |
| *purD*-qPCR-F | 2021 | GCCTAACGCAGTCACACAAA | qRT-PCR | This study |
| *purD*-qPCR-R | 2022 | GCCTAAAAGTGCGGTCAAAA | qRT-PCR | This study |
| *purL*-qPCR-F | 2029 | AGCAGCACCACCACCTAAAC | qRT-PCR | This study |
| *purL*-qPCR-R | 2030 | ACAGGTTCAAAAAGGCGAAA | qRT-PCR | This study |
| *tonB*-qPCR-F | 1414 | GCTACCAAAAGGCGATGAAA | qRT-PCR | (10) |
| *tonB*-qPCR-R | 1415 | TCCGTTCCACTTCCTGCTAC | qRT-PCR | (10) |
| *comC*-qPCR-F | 2013 | ATCGAGGGGAGTTCACGTTT | qRT-PCR | This study |
| *comC*-qPCR-R | 2014 | TTTTCGACTTTCCGACTGCT | qRT-PCR | This study |
| *comD*-qPCR-F | 1827 | GCACAAACAGTAATGGAGCAAA | qRT-PCR | This study |
| *comD*-qPCR-R | 1828 | GAAATCACTACCCCCACCAA | qRT-PCR | This study |
| *comF*-qPCR-F | 2017 | GCAATATCGTAAAGCGTGTGAA | qRT-PCR | This study |
| *comF*-qPCR-R | 2018 | CATCATCCACCAACGCAAC | qRT-PCR | This study |
| *rec2*-qPCR-F | 1829 | ATTGGAAAGCGAAAGAGGTG | qRT-PCR | This study |
| *rec2*-qPCR-R | 1830 | TACCATTGTTGCCGATCAAA | qRT-PCR | This study |
| *purC*-qPCR-F | 2023 | ATAAGCGGTCAGAAGCAACC | qRT-PCR | This study |
| *purC*-qPCR-R | 2024 | TGACACAACAACTCCCCATTT | qRT-PCR | This study |
| *dprA*-qPCR-F | 1833 | TCAAGGTTGTCATCGCCTAA | qRT-PCR | This study |
| *dprA*-qPCR-R | 1834 | CGAGCAACTGAACAAGCAAA | qRT-PCR | This study |
| *comB*-qPCR-F | 1831 | GTAACGCGCAAGATCAAACA | qRT-PCR | This study |
| *comB*-qPCR-R | 1832 | CCCCTTGTTCCACATCAAA | qRT-PCR | This study |
| *hisG*-qPCR-F | 1951 | GGCGAAAATGTGTTAGAAGAGG | qRT-PCR | This study |
| *hisG*-qPCR-R | 1952 | GACGACAATCACCAAAATCAAG | qRT-PCR | This study |
| *comE*-qPCR-F | 2015 | GGTTCTCGTGTCGCTTATGG | qRT-PCR | This study |
| *comE*-qPCR-R | 2016 | CTTCGCTTTTCGTGATTGTG | qRT-PCR | This study |
| *qseC*-qPCR-F | 1955 | AAGGAGAAAATCTGGCGATG | qRT-PCR | This study |
| *qseC*-qPCR-R | 1956 | CATAGCTGAAGTGCGGTCAA | qRT-PCR | This study |
| *purF*-qPCR-F | 2033 | CACAAACTCGAAACCCACAA | qRT-PCR | This study |
| *purF*-qPCR-R | 2034 | AAACGTGAAGAAAATGGCAAA | qRT-PCR | This study |
| *oppA*-qPCR-F | 1953 | GGCTGAATTAGGCGTGGA | qRT-PCR | This study |
| *oppA*-qPCR-R | 1954 | GTTTTCAATGGCGAGGAATG | qRT-PCR | This study |
| *toxA*-qPCR-F | 2019 | CACGCATTGAAAAAGGACAA | qRT-PCR | This study |
| *toxA*-qPCR-R | 2020 | CTTAGAGGCACAATCGACAGG | qRT-PCR | This study |
| *cvpA*-qPCR-F | 2031 | TTTGCGTAAGATAAGTGGCAAG | qRT-PCR | This study |
| *cvpA*-qPCR-R | 2032 | ACTTGGTAGCTGGGTTGTGG | qRT-PCR | This study |
| *pilA*-qPCR-F | 2011 | CCACTATCGCAATTCCCTCTT | qRT-PCR | This study |
| *pilA*-qPCR-R | 2012 | TCCACCCGTACAGTTTGTTG | qRT-PCR | This study |
| *purB*-qPCR-F | 2445 | GGCGTAGGATTAGGTTATTGTTTG | qRT-PCR | This study |
| *purB*-qPCR-R | 2446 | CTTCCCAGTTTTGATTGAGTTCTT | qRT-PCR | This study |
| *glpQ*-qPCR-F | 1959 | CCCAAATGAAATCAGACAAAATC | qRT-PCR | This study |
| *glpQ*-qPCR-R | 1960 | GCAACATCAGTCAAGCCATC | qRT-PCR | This study |
| *pta*-qPCR-F | 1856 | CTACATCAGCACCGCTACCA | qRT-PCR | (11) |
| *pta*-qPCR-R | 1857 | CACCACTGCAAACACCATTC | qRT-PCR | (11) |
| *bamE*-qPCR-F | 2003 | GCCTCGCTTCTTGTTCCAC | qRT-PCR | This study |
| *bamE*-qPCR-R | 2004 | CGCCTCTAAATAGTTGCCTTG | qRT-PCR | This study |
| *hbpA*-qPCR-F2 | 1613 | CTGGATCGTACGGATAATCTTGG | qRT-PCR | (10) |
| *hbpA*-qPCR-R2 | 1614 | AAGGCGCAGGAATATCAGCTAA | qRT-PCR | (10) |
| *gyrA*-qPCR-F2 | 1078 | ATATGTTGGTTGATGGGCAAGG | qRT-PCR | (12) |
| *gyrA*-qPCR-R2 | 1079 | GGCGAGAAATTGACGGTTTCT | qRT-PCR | (12) |
| *mgapdh*-qPCR-F | 1430 | CCCACTAACATCAAATGGGG | qRT-PCR | (13) |
| *mgapdh*-qPCR-R | 1431 | CCTTCCACAATGCCAAAGTT | qRT-PCR | (13) |
| *tnaA*-qPCR-F1 | 2188 | ATGGCTGGCTTATCGTATCG | qRT-PCR | This study |
| *tnaA*-qPCR-R1 | 2189 | AAAGTGCTTGTGCTGGGAAT | qRT-PCR | This study |
| *glyA*-qPCR-F | 2390 | GCAATTGACCGTGCGAAAGA | qRT-PCR | This study |
| *glyA*-qPCR-R | 2391 | TGCGTTGATTAATGCAGGCG | qRT-PCR | This study |
| *aspC*-qPCR-F | 2460 | CTGGTATTGACCCTACTCCAGAAC | qRT-PCR | This study |
| *aspC*-qPCR-R | 2461 | GCCATAAGCATCTTCATCTAATCC | qRT-PCR | This study |
| *thrC*-qPCR-F | 2462 | GATGCTAAGTGATAGCGAAACAGA | qRT-PCR | This study |
| *thrC*-qPCR-R | 2463 | AGGTAATGGAAGCTGAATACCAAG | qRT-PCR | This study |
| *sdaC*-qPCR-F | 2256 | CGCCATATCAGCCATACAGA | qRT-PCR | This study |
| *sdaC*-qPCR-R | 2257 | AGCGGGCCAAAATAAGAGAT | qRT-PCR | This study |
| *purH*-F1 | 1809 | CAGATCCACAAGTGCAACAGCAATTTC | Disruption cassette | This study |
| *purH*-R1 | 1810 | CGTAGCATTATTGGCTGCGTTTCTGGA | Disruption cassette | This study |
| *purH*-F2 | 1811 | TGGCATCTGATGCGTTCTTCCCATTCC | Disruption cassette | This study |
| *purH*-R2 | 1812 | ACCTTGAGCAAACTCTACAATACCCGT | Disruption cassette | This study |
| *comD*-F1 | 1760 | TGGCGTACTTATCAACATCAAAAGCGT | Disruption cassette | This study |
| *comD*-R1 | 1761 | TAGAAATATGTGGCGTCACTTCCAAAC | Disruption cassette | This study |
| *comD*-F2 | 1776 | CAATGTTCAATTTATGCGTAAGCTAGG | Disruption cassette | This study |
| *comD*-R2 | 1777 | TCAAACTGAGAACGGTTACGCTGTGTT | Disruption cassette | This study |
| *rec2*-F1 | 1764 | CACATTTTTATCATAGCGACGTGTTTG | Disruption cassette | This study |
| *rec2*-R1 | 1765 | ACATGCTGCCATTCCACTTTGAAATTG | Disruption cassette | This study |
| *rec2*-F2 | 1780 | CATCGACAAGTGAATACTTGCTTTCTC | Disruption cassette | This study |
| *rec2*-R2 | 1781 | TCTCCAATGTTGCTGTGGCGATAAGCG | Disruption cassette | This study |
| *dprA*-F1 | 1768 | AATGCCTGTACGGCCTGCATCTTCGCA | Disruption cassette | This study |
| *dprA*-R1 | 1769 | CCAGCGTATCTGGTTTAGCAATATACA | Disruption cassette | This study |
| *dprA*-F2 | 1784 | GTTACACGCCAGTGAGCATTGATGATT | Disruption cassette | This study |
| *dprA*-R2 | 1785 | CAACATCATCATAATTAAGTAATTCAT | Disruption cassette | This study |
| *comB*-F1 | 1766 | AGAGACCTATGATTTGGCTGTTAAGTA | Disruption cassette | This study |
| *comB*-R1 | 1767 | CTATCACATTGCTCTCCTAGCTTACGC | Disruption cassette | This study |
| *comB*-F2 | 1782 | GTCAGGTTCAACCTGAACAAGATACAT | Disruption cassette | This study |
| *comB*-R2 | 1783 | GCTAAAAGCCAACATTAAATTAATAGC | Disruption cassette | This study |
| *comE*-F1 | 1770 | GTTCAACCTGAACAAGATACATTGTTT | Disruption cassette | This study |
| *comE*-R1 | 1771 | CACATTCCGCATTAGGCGTACCAGGAT | Disruption cassette | This study |
| *comE*-F2 | 1786 | CTATTGATAAACAAGAAATTAATACTC | Disruption cassette | This study |
| *comE*-R2 | 1787 | TATATCTCCAATCACTAAATTCACATC | Disruption cassette | This study |
| *qseBC*-F1 | 1937 | AGGCGAGGGAATTTCCCAAGAT | Disruption cassette | This study |
| *qseBC*-R1 | 1938 | CGCTAAACACATCACCACAACA | Disruption cassette | This study |
| *qseBC*-F2 (2) | 2045 | TGCGAATTGCGGAATTACACGAA | Disruption cassette | This study |
| *qseBC*-R2 (2) | 2046 | CAAAACCAAGTTTAGTTAAACCA | Disruption cassette | This study |
| *glpQ*-F1 | 1575 | ATTTTCTTCAGCACTCACGGTATTG | Disruption cassette | This study |
| *glp*Q-R1 | 1572 | TAAATTGCCGCCAATTGCTTTTTTG | Disruption cassette | This study |
| *glpQ* -F2 | 1576 | CTAAAAACCTCATAAAAATTTACCGCACTCTCAAGGAGAAAATACTTATGATTCCGGGGATCCGTCGACC | Disruption cassette | This study |
| *glpQ*-R2 | 1577 | GGTATGTTTACCTACGGTTATAATGAAATTATTTTATTCCTTTTAAGAATGTAGGCTGGAGCTGCTTCG | Disruption cassette | This study |

**Table S3.** Determination of transformation frequency of NTHi375 WT and mutant strains, in MIV medium, by using MAP-7 gDNA.

| **Strain** | **Transformation frequency** |
| --- | --- |
| NTHi375 | 3 x 10^-5^ |
| Δ*comB* | 10^-9^- 0 |
| Δ*comD* | 0 |
| Δ*comE* | 0 |
| Δ*rec2* | 0 |
| Δ*dprA* | 10^-7^- 10^-8^ |

**
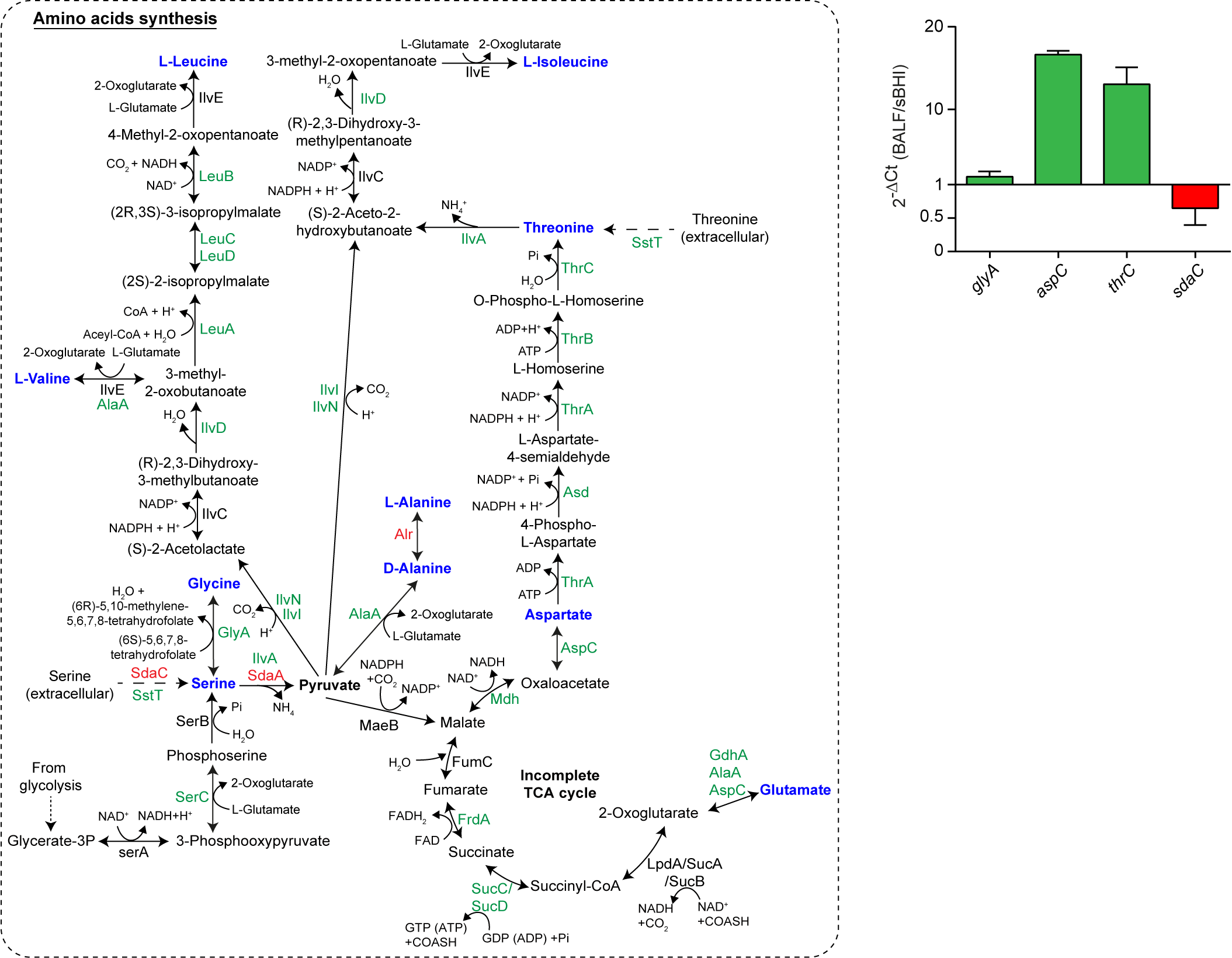
**

**Figure S1. *H. influenzae* differentially expressed genes involved in amino acid synthesis (NTHi sBHi *vs* BALF samples).** Green, reactions catalyzed by genes up-regulated *in vivo*; red, reactions catalyzed by genes down-regulated *in vivo*. Amino acids are indicated in blue.

Right panel: RT-qPCR validation of selected *H. influenzae* differentially expressed genes, up- (green) or down-regulated (red) *in vivo*. NTHi375 was grown in sBHI and collected in exponential phase; BALF samples were recovered from NTHi375-infected mice at 12 hpi. Purified RNA was used to determine the ratio of bacterial gene expression by qRT-PCR. Data are shown as mean ± SD.

**
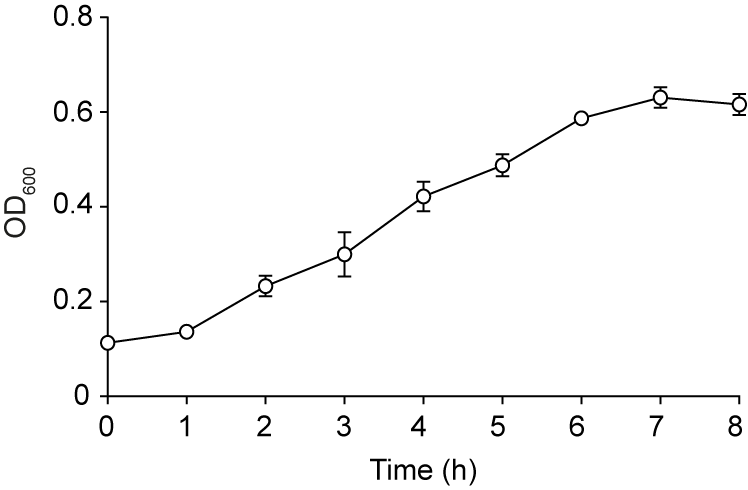
**

**Figure S2. Growth of *H. influenzae* NTHi375 in sASM.** sBHI overnight grown cultures were diluted in sASM to OD_600_=0.1, and grown in 25 mL sASM and shaking. OD_600_ were recorded every 1 h for up to 8 h. Approximately, 2.5x10^8^ CFU/mL were plated when cultures reached OD_600_=0.3; at this time point, samples were used for RNA extraction.

**
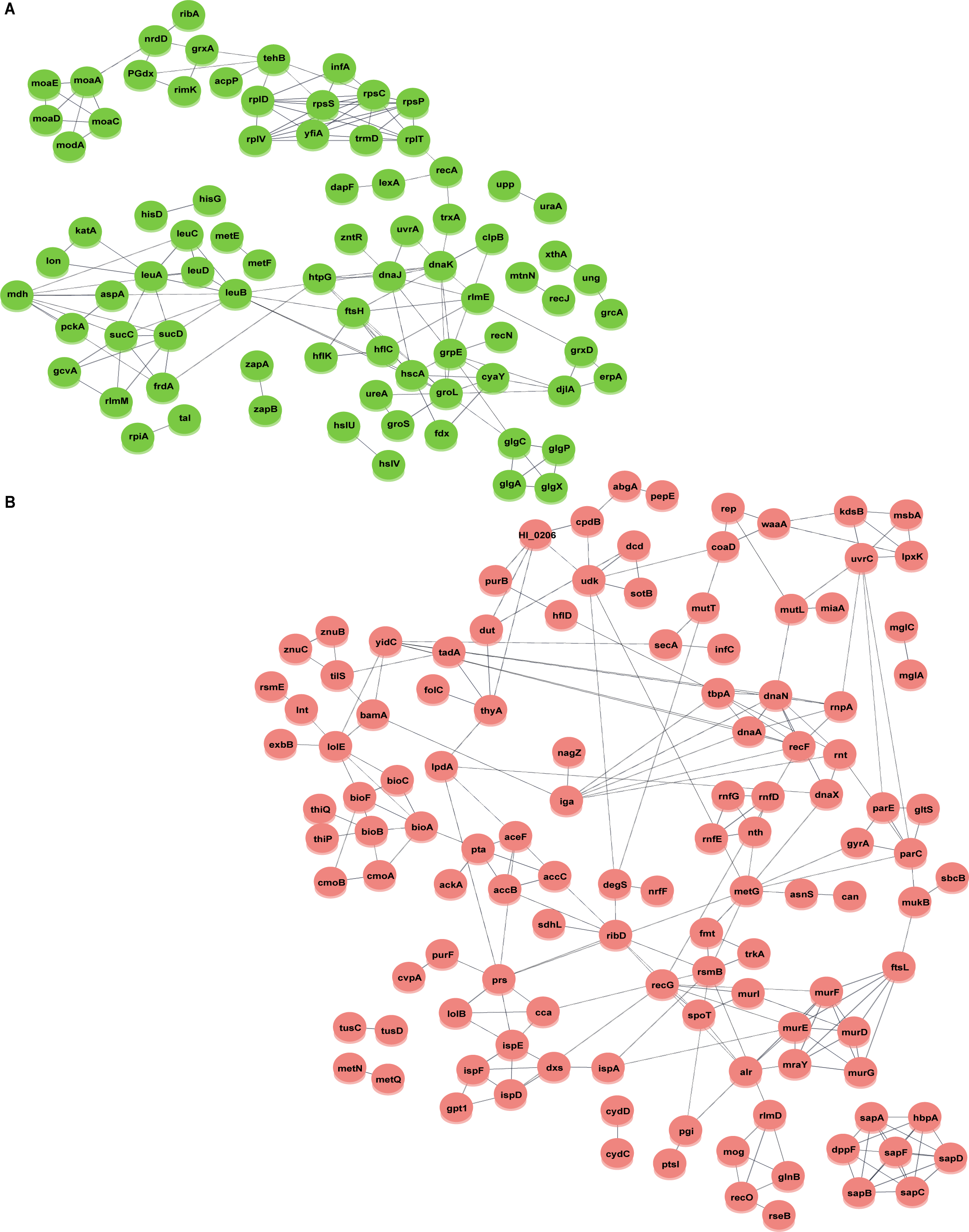
**

**Figure S3. STRING analyses of *H. influenzae* genes differentially expressed in sBHI compared to sASM samples.** Up-regulated (A) and down-regulated (B) genes. The node colors are based on the dataset's differential regulation of each gene (up-regulated: green, down-regulated: red).

**References**

1. Allen S, Zaleski A, Johnston JW, Gibson BW, Apicella MA. 2005. Novel sialic acid transporter of *Haemophilus influenzae*. Infect Immun 73:5291–5300.

2. Tracy E, Ye F, Baker BD, Munson RSJ. 2008. Construction of non-polar mutants in *Haemophilus influenzae* using FLP recombinase technology. BMC Mol Biol 9:101.

3. Herriott RM, Meyer EM, Vogt M. 1970. Defined nongrowth media for stage II development of competence in *Haemophilus influenzae*. J Bacteriol 101:517–524.

4. Poje G, Redfield RJ. 2003. Transformation of *Haemophilus influenzae*. Methods Mol Med 71:57–70.

5. Mell JC, Viadas C, Moleres J, Sinha S, Fernández-Calvet A, Porsch EA, St. Geme JW, Nislow C, Redfield RJ, Garmendia J. 2016. Transformed recombinant enrichment profiling rapidly identifies HMW1 as an intracellular invasion locus in *Haemophilus influenzae*. PLoS Pathog 12:e1005576.

6. Bouchet V, Hood DW, Li J, Brisson JR, Randle GA, Martin A, Li Z, Goldstein R, Schweda EK, Pelton SI, Richards JC, Moxon ER. 2003. Host-derived sialic acid is incorporated into *Haemophilus influenzae* lipopolysaccharide and is a major virulence factor in experimental otitis media. Proc Natl Acad Sci U S A 100:8898–8903.

7. Martí-Lliteras P, Regueiro V, Morey P, Hood DW, Saus C, Sauleda J, Agustí AGN, Bengoechea JA, Garmendia J. 2009. Nontypeable *Haemophilus influenzae* clearance by alveolar macrophages is impaired by exposure to cigarette smoke. Infect Immun 77:4232–4242.

8. Morey P, Viadas C, Euba B, Hood DW, Barberan M, Gil C, Grillo MJ, Bengoechea JA, Garmendia J. 2013. Relative contributions of lipooligosaccharide inner and outer core modifications to nontypeable *Haemophilus influenzae* pathogenesis. Infect Immun 81:4100–4111.

9. Whitby PW, VanWagoner TM, Seale TW, Morton DJ, Stull TL. 2013. Comparison of transcription of the *Haemophilus influenzae* iron/heme modulon genes *in vitro* and *in vivo* in the chinchilla middle ear. BMC Genomics 14:925.

10. Rodríguez-Arce I, Al-Jubair T, Euba B, Fernández-Calvet A, Gil-Campillo C, Martí S, Törnroth-Horsefield S, Riesbeck K, Garmendia J. 2019. Moonlighting of *Haemophilus influenzae* heme acquisition systems contributes to the host airway-pathogen interplay in a coordinated manner. Virulence 10:315–333.

11. López-López N, Euba B, Hill J, Dhouib R, Caballero L, Leiva J, Hosmer J, Cuesta S, Ramos-Vivas J, Díez-Martínez R, Schirra HJ, Blank LM, Kappler U, Garmendia J. 2020. *Haemophilus influenzae* glucose catabolism leading to production of the immunometabolite acetate has a key contribution to the host airway-pathogen interplay. ACS Infect Dis 6:406–421.

12. Moleres J, Fernández-Calvet A, Ehrlich RL, Martí S, Pérez-Regidor L, Euba B, Rodríguez-Arce I, Balashov S, Cuevas E, Liñares J, Ardanuy C, Martín-Santamaría S, Ehrlich GD, Mell JC, Garmendia J. 2018. Antagonistic pleiotropy in the bifunctional surface protein FadL (OmpP1) during adaptation of *Haemophilus influenzae* to chronic lung infection associated with chronic obstructive pulmonary disease. MBio 9:1–23.

13. Regueiro V, Moranta D, Frank CG, Larrarte E, Margareto J, March C, Garmendia J, Bengoechea JA. 2011. *Klebsiella pneumoniae* subverts the activation of inflammatory responses in a NOD1-dependent manner. Cell Microbiol 13:135–153.
